# Cell-free protein synthesis from genomically recoded bacteria enables multisite incorporation of noncanonical amino acids

**DOI:** 10.1038/s41467-018-03469-5

**Published:** 2018-03-23

**Authors:** Rey W. Martin, Benjamin J. Des Soye, Yong-Chan Kwon, Jennifer Kay, Roderick G. Davis, Paul M. Thomas, Natalia I. Majewska, Cindy X. Chen, Ryan D. Marcum, Mary Grace Weiss, Ashleigh E. Stoddart, Miriam Amiram, Arnaz K. Ranji Charna, Jaymin R. Patel, Farren J. Isaacs, Neil L. Kelleher, Seok Hoon Hong, Michael C. Jewett

**Affiliations:** 1 Department of Chemical and Biological Engineering, Northwestern University, Evanston, Illinois 60208 USA; 2 Chemistry of Life Processes Institute, Northwestern University, Evanston, Illinois 60208 USA; 3 Center for Synthetic Biology, Northwestern University, Evanston, Illinois 60208 USA; 4 Interdisciplinary Biological Sciences Program, Northwestern University, Evanston, Illinois 60208 USA; 5 Department of Biological and Agricultural Engineering, Louisiana State University, Baton Rouge, Louisiana 70803 USA; 6 Proteomics Center of Excellence, Northwestern University, Evanston, Illinois 60208 USA; 7 Department of Molecular Biosciences, Northwestern University, Evanston, Illinois 60208 USA; 8 Robert H. Lurie Comprehensive Cancer Center, Northwestern University, Chicago, Illinois 60611 USA; 9 Department of Molecular Cellular, and Developmental Biology, Yale University, New Haven, Connecticut 06520 USA; 10 Systems Biology Institute, Yale University, New Haven, Connecticut 06516 USA; 11 Department of Chemistry, Northwestern University, Evanston, Illinois 60208 USA; 12 Department of Chemical and Biological Engineering, Illinois Institute of Technology, Chicago, Illinois 60616 USA; 13 Simpson Querrey Institute, Northwestern University, Chicago, Illinois 60611 USA

## Abstract

Cell-free protein synthesis has emerged as a powerful approach for expanding the range of genetically encoded chemistry into proteins. Unfortunately, efforts to site-specifically incorporate multiple non-canonical amino acids into proteins using crude extract-based cell-free systems have been limited by release factor 1 competition. Here we address this limitation by establishing a bacterial cell-free protein synthesis platform based on genomically recoded *Escherichia coli* lacking release factor 1. This platform was developed by exploiting multiplex genome engineering to enhance extract performance by functionally inactivating negative effectors. Our most productive cell extracts enabled synthesis of 1,780 ± 30 mg/L superfolder green fluorescent protein. Using an optimized platform, we demonstrated the ability to introduce 40 identical *p*-acetyl-l-phenylalanine residues site specifically into an elastin-like polypeptide with high accuracy of incorporation ( ≥ 98%) and yield (96 ± 3 mg/L). We expect this cell-free platform to facilitate fundamental understanding and enable manufacturing paradigms for proteins with new and diverse chemistries.

## Abstract (web summary)

Cell-free protein synthesis allows for producing proteins without the need of a host organism, thus sparing the researcher experimental hassle. Here, the authors developed a cell-free synthesis method that enables incorporating non-standard amino acids in the product.

## Introduction

Cell-free synthetic biology is emerging as a transformative approach to understand, harness, and expand the capabilities of natural biological systems^1^. The foundational principle is that complex biomolecular transformations are conducted without using intact cells. Instead, crude cell lysates (or extracts) are used, which provides a unique freedom of design to control biological systems for a wide array of applications. For example, cell-free protein synthesis (CFPS) systems have been used to decipher the genetic code^2^, prototype genetic circuits and metabolic pathways^3–7^, enable portable diagnostics^8^, facilitate on-demand biomolecular manufacturing^9,10^, and produce antibody therapeutics at the commercial scale^11^. The recent surge of applications has revitalized interest in cell-free systems, especially in areas where limits imposed by the organism may impede progress. One such area is expanding the genetic code to incorporate non-canonical amino acids (ncAAs) into proteins, where the extent of engineering can be limited by the fitness of the organism^12–15^.

Pioneering efforts by Schultz and others have demonstrated it is possible to genetically encode more than 150 ncAAs into proteins, and that this encoding can be a powerful tool^15,16^. For example, site-specific incorporation of ncAAs at single positions in proteins have provided new ways to study protein structure, dynamics, and posttranslational modifications^17^, as well as manufacture protein–drug conjugates^18,19^. However, inefficiencies associated with the engineered orthogonal translation (TL) machinery (e.g., TL elements that specifically use a ncAA and do not interact with the cell’s natural TL apparatus) have limited the ability to incorporate multiple ncAAs into proteins with high purity and yields^20,21^. A key constraint is that codon re-assignment strategies typically rely on amber suppression^22^, where the amber UAG stop codon is re-assigned to encode a ncAA and the orthogonal transfer RNA anticodon is mutated to CUA. The orthogonal ncAA-tRNA_CUA_ must then outcompete essential TL machinery (e.g., release factor 1, RF1) for the UAG codon. Historically, this competition has led to poor protein expression yields, as premature termination by RF1 exponentially increases with the number of amber codons in the coding sequence^23^. Poor protein expression yields limit applications in both basic and applied science.

Recently, a genomically recoded *Escherichia* *coli* strain was developed (*C321*.*∆A*) in which all 321 occurrences of the UAG stop codon were reassigned to the synonymous UAA codon^24^ using multiplex automated genome engineering (MAGE)^25^ and conjugative assembly genome engineering (CAGE)^26^. This allowed for the genomic deletion of RF1 (i.e., *∆prfA* or *∆A*) without affecting cellular physiology, thus freeing the UAG codon for dedicated ncAA incorporation^24^. Precursor RF1-deficient strains in which only a small set of essential genes were recoded have already shown the potential to produce proteins with improved ncAA incorporation efficiencies as compared with strains with RF1^24,27^; however, the upregulation of natural suppression mechanisms (e.g., *ssrA*) is problematic, because they promote the formation of truncation products, especially for tens of incorporation events^24,27,28^. The fully recoded *C321*.*∆A* strain avoids these problems and we recently showed the possibility of using *C321*.*∆A* coupled with extensively engineered synthetases for multi-site incorporation of up to 30 ncAAs into a single biopolymer in vivo^20^. Based on these results, we hypothesized that the fully recoded *C321*.*∆A* strain would serve as an ideal chassis strain for the development of crude extract-based cell-free systems capable of highly efficient, multi-site ncAA incorporation into biopolymers. Such a system would complement in vivo manufacturing strategies, with some advantageous features^29,30^. For example, the open reaction environment means the supply of orthogonal TL system (OTS) components and their substrates necessary for high-level ncAA incorporation can be provided and controlled at precise ratios as a way to overcome enzyme inefficiencies. In addition, cell-free systems are not limited by viability requirements, thus avoiding constraints arising from toxic OTS components^27^. Our proposed approach based on the *C321*.*∆A* strain might also provide cost and ease of use advantages over other cell-free systems that have tried to reduce the effects of RF1 competition by using reconstituted systems^31^, antibody inhibitors^32^, RF1 depletion by subtractive chromatography^33^, or partially recoded *E*. *coli* strains with elevated natural suppression mechanisms^34^.

Here we describe the development of a CFPS platform from the genomically recoded *C321*.*∆A* to manufacture proteins with tens of identical site specifically introduced ncAAs. Specifically, we use MAGE to improve protein production capacity by inactivating negative effectors in the host strain such that they are not present in the lysate. By testing tens of strain variants, we isolate a CFPS platform capable of synthesizing 1,780 ± 30 mg/L of superfolder green fluorescent protein (sfGFP), as well as modified sfGFP containing up to five *p*-acetyl-l-phenylalanine (pAcF) residues at high purity ( ≥ 98%). Using an optimized CFPS platform, we test the ability to synthesize elastin-like polypeptides (ELPs) that contain up to 40 UAG codons. We demonstrate incorporation of 40 ncAAs per ELP protein with high yields (~ 100 mg/L) and high fidelity ( ≥ 98%) of site-specific ncAA incorporation.

## Results

### CFPS from extracts of a genomically recoded organism

**Fig. 1:**
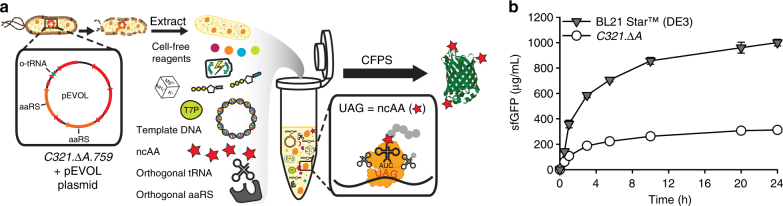
CFPS from extracts of a genomically recoded organism. **a** Schematic of the production and utilization of crude extract from genomically recoded organisms with plasmid overexpression of orthogonal translation components for cell-free protein synthesis (CFPS). CFPS reactions are supplemented with the necessary substrates (e.g., amino acids, NTPs, etc.) required for in vitro transcription and translation as well as purified orthogonal translation system (OTS) components to help increase the ncAA incorporation efficiency. aaRS, aminoacyl tRNA synthetase; ncAA, non-canonical amino acid; T7P, T7 RNA polymerase; UAG, amber codon. **b** Time course of superfolder green fluorescent protein (sfGFP) synthesis catalyzed by extracts derived from a genomically recoded organism, *C321*.*∆A*, and a commercial strain, BL21 Star (DE3). Three independent batch CFPS reactions (*n* = 3) were performed at 30 °C for each time point over 24 h. Error bar = 1 SD

To benchmark CFPS activity, we first compared sfGFP yields in extracts from *C321*.*∆A* and BL21 Star (DE3), the standard commercial protein expression strain (Fig. 1a). Combined transcription (TX)–TL reactions were carried out in 15 µL volumes for 24 h at 30 °C. Protein yields from BL21 Star (DE3) extracts were > 3-fold higher than those from *C321*.*∆A* (Fig. 1b), highlighting the need to improve protein synthesis yields to take advantage of the benefits of RF1 removal for making modified proteins with ncAAs for preparative purposes.

Previously, genomic modifications to the extract source strain to stabilize DNA template^35^, amino acid supply^36^, and protein degradation^37^ have improved CFPS yields from other source strains. For example, we engineered a partially recoded strain of *E*. *coli* (*rEc*.*E13*.*∆A*) by disrupting genes encoding nucleases (MCJ.559 (*endA*^−^ *csdA*^−^)) to improve protein synthesis yields > 4-fold relative to the parent strain^34^. Building on this knowledge, we hypothesized that the genomic disruption of negative protein effectors in *C321*.*∆A* extracts would help stabilize essential substrates in cell-free reactions, extend reaction durations, and increase CFPS yields.

### Strain engineering for improved CFPS performance

**Fig. 2:**
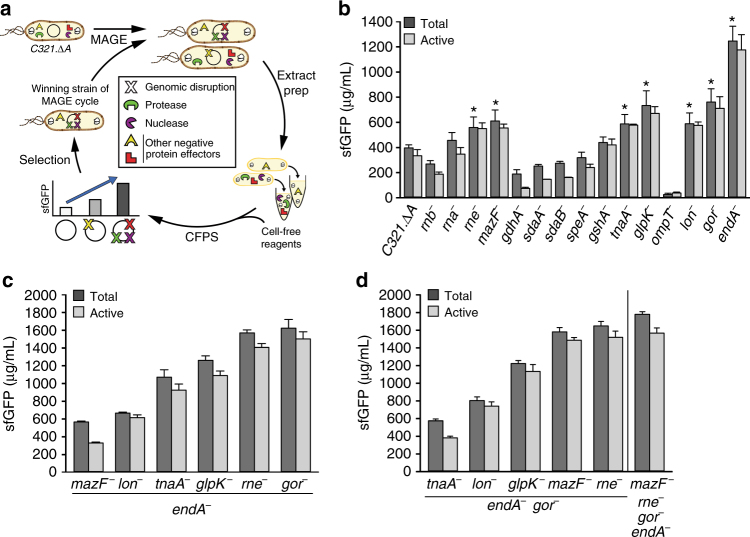
Engineering *C321*.*∆A* variants for enhanced CFPS. **a** Schematic of design-build-test cycles employing multiplex automated genome engineering (MAGE) to disrupt putative negative protein effectors (Supplementary Table 1) in engineered *C321*.*∆A* strains for producing extracts with enhanced cell-free protein synthesis (CFPS) yields. **b** Cell extracts derived from *C321*.*∆A* and genomically engineered strains containing a single putative negative effector inactivation were screened for sfGFP yields. Beneficial mutations that increase active yields ≥ 50% relative to C321.ΔA are highlighted with an *(*p* < 0.01, Student’s *t*-test). **c** *C321*.*∆A*.*542* (*endA*^−^) was chosen as the next base strain and the following beneficial disruptions were pursued in combination: *rne*, *mazF*, *tnaA*, *glpK*, *lon*, and *gor*. **d** *C321*.*∆A*.*709* (*endA*^−^ *gor*^−^) was selected as the subsequent base strain for triple and quadruple mutant construction. *C321*.*∆A*.*759* (*endA*^−^ *gor*^−^ *rne*^−^ *mazF*^−^) yielded the highest level of CFPS production. Total sfGFP concentration was measured by counting radioactive ^14^C-Leucine incorporation and active protein was measured using fluorescence. Three independent batch CFPS reactions were performed for each sample at 30 °C for 20 h (*n* = 3). Error bar = 1 SD

We targeted the functional inactivation of five nucleases (*rna*, *rnb*, *mazF*, *endA*, and *rne*), two proteases (*ompT* and *lon*), and eight targets shown previously to negatively impact amino acid, energy, and redox stability (*gdhA*, *gshA*, *sdaA*, *sdaB*, *speA*, *tnaA*, *glpK*, and *gor*) in *C321*.*∆A* individually and in combination (Supplementary Table 1). Our effort followed a five-step approach. First, we generated a library of single mutant strains in which we used MAGE to insert an early TL termination sequence into the open reading frames of gene targets that would functionally inactivate them, as we have done before^34^ (Fig. 2a and Supplementary Tables 2 and 3). Second, we confirmed gene disruptions using multiplex allele specific PCR and DNA sequencing. Third, we measured the growth rate for each of the MAGE-modified strains, noting that average doubling time increased 9 ± 9% above the parent strain (Supplementary Table 4). Fourth, cell extracts from each strain were generated using a high-throughput and robust extract generation procedure^38^. Fifth, we tested the strains in CFPS to assess their overall protein synthesis capability. We observed that seven single functional inactivation mutations increased CFPS yields more than 50% relative to the wild type *C321*.*∆A* strain; namely, *rne*^−^, *mazF*^−^, *tnaA*^−^, *glpK*^−^, *lon*^−^, *gor*^−^, and *endA*^−^ (Fig. 2b). These results suggested that some of the protein effectors targeted for inactivation were deleterious to CFPS activity. They also demonstrated the difficulty associated with predicting CFPS productivities from engineered strains. For example, some mutations identified in previous screens (e.g., *rnb*^−^ in *rEc*.*E13*.*∆A*)^34^ were not beneficial in the *C321*.*∆A* context, others which reduced cellular fitness enhanced CFPS activity (e.g., *lon*^−^), and yet others with no impact on cell growth (e.g., *ompT*^−^) led to poor extract performance (Fig. 2b).

With improvements in hand from single mutant strains, we next set out to identify synergistic benefits to CFPS productivity by combining highly productive mutations. We introduced the *rne*^−^, *mazF*^−^, *tnaA*^−^, *glpK*^−^, *lon*^−^, and *gor*^−^ mutations to the best performing strain from our initial screen, strain *C321*.*∆A*.*542* (*endA*^−^) (Fig. 2c). The combination of *endA*^−^ and *gor*^−^ mutations resulted in an extract capable of synthesizing 1,620 ± 10 mg/L of active sfGFP (strain *C321*.*∆A*.*709*). We then used *C321*.*∆A*.*709* to generate six additional strains with combined mutations. Although we did not observe synergistic enhancements, our top performing extract chassis strain (*C321*.*∆A*.*759* (*endA*^−^ *gor*^−^ *rne*^−^ *mazF*^−^)) resulted in total yields of 1,780 ± 30 mg/L (Fig. 2d), representing a 4.5-fold increase in sfGFP yield relative to the progenitor strain (*C321*.*∆A*). In addition, we tested 12 combinatorial mutants generated throughout our MAGE screening, and although a few demonstrated CFPS yields > 1 g/L of active sfGFP, none surpassed the CFPS yields observed from *C321*.*∆A*.*759* (Supplementary Table 5). Lastly, we determined that CFPS improvements seen in *C321*.*∆A*.*759* brought on by genomic modifications could not be obtained by simply supplementing *C321*.*∆A*-based reactions with RNAse inhibitors (Supplementary Fig. 1). Final strains were fully sequenced to verify functional targeted modifications in the genome. Whole-genome sequences for strains *C321*.*∆A*, *C321*.*∆A*.*542*, *C321*.*∆A*.*705*, *C321*.*∆A*.*709*, *C321*.*∆A*.*740*, and *C321*.*∆A*.*759* have been deposited in the NCBI SRA collection under accession code PRJNA361365. Each of the targeted mutations were achieved. MAGE has been shown to induce mutations throughout the genome before, and we observed a number of accumulated polymorphisms in the extract chassis strains. These polymorphisms, along with a specific list of protein-coding genes bearing mutations, are shown in Supplementary Tables 6 and 7. In the future, we seek to better understand the systems impact of the non-targeted mutations.

Based on our previous studies using *rEc*.*E13*.*∆A*[34], we hypothesized that the beneficial mutations in *C321*.*∆A*.*759* reduced messenger RNA degradation and stabilized the DNA template. To test mRNA stability, we performed TL-only reactions using extracts derived from *C321*.*∆A*.*759* and *C321*.*∆A*. Purified mRNA template coding for sfGFP was used to direct protein synthesis. We observed a twofold increase in mRNA and ~ 90% increase of active sfGFP using *C321*.*∆A*.*759* extracts relative to *C321*.*∆A* extracts after a 120 min cell-free reaction (Supplementary Fig. 2). To test DNA stability, TX-only reactions were used. Specifically, plasmid DNA containing the modified red fluorescent protein–Spinach aptamer gene (Supplementary Table 3) was pre-incubated with cell extract and a fluorophore molecule, 3,5-difluoro-4-hydroxybenzylidene imidazolinone (DFHBI), for 0, 60, and 180 min. Then, CFPS reagents were added and mRNA was synthesized, then quantified by measuring the fluorescence of DFHBI-bound Spinach aptamer mRNA. After 180 min of pre-incubation, nearly 50% of Spinach aptamer mRNA was synthesized in *C321*.*∆A*.*759* (*endA*^−^) extracts relative to the 0 min control. In contrast, the extract with endonuclease I (*C321*.*∆A*) decreased the maximum mRNA synthesis level by ~ 75% (Supplementary Fig. 3). Together, our data support the hypothesis that inactivating nucleases in the extract chassis strain stabilized DNA and mRNA to improve CFPS yields.

In addition to confirming added DNA and mRNA stability, we also assessed potential changes in energy and amino acid substrate stability that may have occurred in *C321*.*∆A*.*759*– relative to *C321*.*∆A*–based CFPS. Similar trends in ATP levels (Supplementary Fig. 4), adenylate charge (Supplementary Fig. 5), and amino acid concentrations (Supplementary Fig. 6) were observed in CFPS reactions derived from both strains. Supplemental feeding with the amino acids found to be most rapidly depleted did not improve yields (Supplementary Fig. 6f). The similar amino acid and energy stability profiles in *C321*.*∆A*.*759* compared with *C321*.*∆A* suggest that our strain engineering efforts did not modulate the availability of these substrates.

To generalize CFPS improvements in *C321*.*∆A*.*759*, we next expressed four model proteins that have been previously synthesized in CFPS systems and compared productivities to BL21 Star (DE3). We observed a 31–63% increase in soluble and total protein synthesis of sfGFP, chloramphenicol acetyltransferase (CAT), dihydrofolate reductase (DHFR), and modified murine granulocyte-macrophage colony-stimulating factor (mGM-CSF) in our engineered *C321*.*∆A*.*759* extracts as compared to BL21 Star (DE3) extracts (Supplementary Fig. 7a). Autoradiograms of proteins produced using *C321*.*∆A*.*759* extract show production of full-length sfGFP, CAT, DHFR, and mGM-CSF (Supplementary Fig. 7b and 7c). In addition, we observed disulfide bond formation in the model mGM-CSF under an oxidizing CFPS environment (– DTT), as has been previously shown (Supplementary Fig. 7c)^39,40^. In sum, the development of enhanced extract source strains by MAGE enabled a general and high-yielding CFPS platform.

### Multi-site ncAA incorporation into proteins in CFPS

**Fig. 3:**
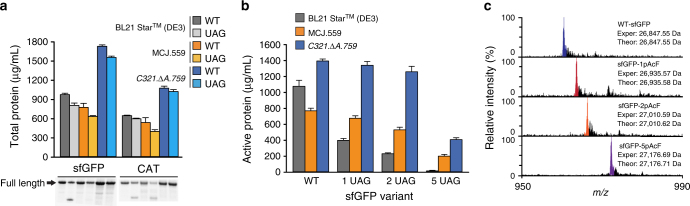
Multi-site incorporation of pAcF into proteins. Cell-free *p*-acetyl-l-phenylalanine (pAcF) incorporation was compared using extracts derived from BL21 Star (DE3), MCJ.559, and *C321*.*∆A*.*759* strains containing the pEVOL-pAcF vector. The pEVOL-pAcF vector harbors the orthogonal translation machinery necessary for pAcF incorporation. **a** Total protein yields for wild-type (WT) and 1 UAG versions of superfolder green fluorescent protein (sfGFP) and chloramphenicol acetyl transferase (CAT) are shown along with an autoradiogram of the resulting protein product. Supplementary Fig. 8 shows the entirety of the autoradiogram along with a molecular weight marker. **b** Multi-site incorporation of pAcF into sfGFP variants as quantified by active protein produced. The sfGFP variants used were wild-type (WT), sfGFP containing a single pAcF corresponding to the position of T216 (1 UAG), sfGFP containing sfGFP containing two pAcFs (2 UAG), and sfGFP containing five pAcFs (5 UAG). Three independent batch CFPS reactions were performed for each sample at 30 °C for 20 h (*n* = 3). Error bar = 1 SD. **c** Spectrum of the 28 + charge state of sfGFP, obtained by top-down mass spectrometry and illustrating site-specific incorporation of pAcF at single and multiple sites. Experimental (Exper) and theoretical (Theor) mass peaks for each sfGFP variant are shown. Major peaks (color) in each spectrum coincide with the theoretical peaks for each species (see also Supplementary Fig. 11). Smaller peaks immediately to the right of the major peaks are due to oxidation of the protein, a common electrochemical reaction occurring during electrospray ionization. Experimentally determined masses are ≤ 1 p.p.m. in comparison of theoretical mass calculations. Owing to the size of pAcF, misincorporation would result in peaks present at lower m/z values relative to the colored theoretical peak

We next aimed to test site-specific ncAA incorporation into proteins using our high-yielding CFPS platform from *C321*.*∆A*.*759*-derived extracts and compare these results to reactions using extracts from BL21 Star (DE3) (containing RF1) and a partially recoded RF1-deficient engineered strain MCJ.559 based on *rEc*.*E13*.*∆A*. To do so, we transformed each organism with pEVOL-pAcF plasmid that expresses both orthogonal pAcF synthetase (pAcFRS) and tRNA (o-tRNA^opt^)^41^. Then, we quantitatively assessed the incorporation of pAcF into sfGFP variants with up to five in-frame amber codons. CFPS reactions were supplemented with additional OTS components based on our previous work^27^. Specifically, we added 10 µg/mL of linear DNA encoding optimized orthogonal tRNA in the form of a transzyme (o-tRNA^opt^) for in situ synthesis of the tRNA. The orthogonal pAcFRS was overproduced, purified as previously described, and added at a level of 0.5 mg/mL. The ncAA, in this case pAcF, was supplied at a level of 2 mM in each CFPS reaction. Total protein yields were quantified by ^14^C-leucine radioactive incorporation. Production of wild-type and modified sfGFP containing one UAG codon (sfGFP-UAG) was increased 77% and 92% in *C321*.*∆A*.*759* extracts as compared with BL21 Star (DE3), and 120% and 145% as compared with MCJ.559, respectively (Fig. 3a and Supplementary Fig. 8a). Moreover, we observed that sfGFP-UAG was expressed at 90% the level of wild-type sfGFP. Owing to the absence of RF1 competition, the major protein produced was full-length sfGFP using extracts derived from *C321*.*∆A*.*759* and MCJ.559, whereas truncated sfGFP was visible in reactions catalyzed by BL21 Star (DE3) extract, presumably due to RF1 competition (Fig. 3a and Supplementary Fig. 8a)^30,42^. Similar results were obtained with a second model protein, CAT with an in-frame amber codon at position 112 (CAT-UAG) (Fig. 3a and Supplementary Fig. 8b). When expressing CAT-UAG using MCJ.559 extract, similar levels of truncated CAT relative to BL21 Star (DE3) were observed; however, this is most likely due to an upregulation of rescue mechanisms for ribosome stalling in the partially recoded strain^34^. Single pAcF incorporation into CAT-UAG using *C321*.*∆A*.*759* lysate demonstrated only full-length product. Therefore, our completely recoded, genomically engineered *C321*.*∆A*.*759* strain provides benefits for efficient ncAA incorporation without detectable levels of truncation product.

We then evaluated the ability of our high-yielding CFPS platform to facilitate incorporation of up to five identical ncAAs into sfGFP. For ease of analysis, a fluorescence assay was used, which indicated increased production of sfGFP in extracts from *C321*.*∆A*.*759* (Fig. 3b). Results for BL21 Star (DE3) extract displayed an exponential decrease in active sfGFP synthesized with an increasing presence of UAG, leading to the production of no detectable active protein for sfGFP-5UAG. Active protein produced by *C321*.*∆A*.*759* extract were ~ 2-fold greater than that produced by MCJ.559 extract, suggesting that benefits observed in increased yield can be extended to multi-site ncAA incorporation for our enhanced, fully recoded strain. Furthermore, we examined the ability to incorporate consecutive pAcFs into single protein. Protein gel and autoradiogram analysis of sfGFP with eight and nine consecutive amber codons indicated that this is possible, with the percent of full-length product being ~ 75% and 60%, respectively (Supplementary Fig. 9).

In addition, batch reactions catalyzed by *C321*.*∆A*.*759* extracts could also be scaled 17-fold without loss of productivity provided that a proper ratio of surface area to volume ratio is maintained (Supplementary Fig. 10)^43^. Of note, we believe our reactions could be further scaled to a wide range of volumes to produce larger amounts of protein if accounting for surface area to volume effects. For example, Sutro Biopharma has applied *E*. *coli*-based CFPS platforms to clinical manufacturing of therapeutics at the 100 L scale^44^, with an expansion factor of 10^6^. In terms of cost, although we use a phosphoenolpyruvate (PEP)-based CFPS system here, cellular metabolism could be used to fuel cost effective, high-level protein synthesis suitable for manufacturing applications^45,46^.

After demonstrating benefits for protein expression, we carried out top-down mass spectrometry (i.e., MS analysis of whole intact proteins) to detect and provide semi-quantitative data for the incorporation efficiency of pAcF into sfGFP using extract derived from *C321*.*∆A*.*759*. Figure 3c shows the 28 + charge state of sfGFP and clearly illustrates mass shifts corresponding to the incorporation of one, two, and five pAcF residues. Site-specific incorporation of pAcF, as detected by MS, was ≥ 98% in all samples, with ≤ 1 p.p.m. difference between experimental and theoretical protein masses (Supplementary Fig. 11). In other words, efficient and high yielding site-specific pAcF incorporation into sfGFP was observed when using *C321*.*∆A*.*759* extract. We went on to further show that extracts generated from *C321*.*∆A*.*759* are compatible with multiple OTSs, showing the incorporation of *p*-propargyloxy-l-phenylalanine and *p*-azido-l-phenylalanine (pAzF) (Supplementary Fig. 12).

### Multi-site ncAA incorporation into ELPs

We next explored the synthesis of sequence-defined biopolymers containing tens of site specifically introduced ncAAs using our efficient and tunable CFPS system. As a model biopolymer, we selected ELPs. ELPs are biocompatible and stimuli-responsive biopolymers that can be applied for drug delivery and tissue engineering^47,48^. Typically, ELPs consist of repeats of the pentapeptide sequence VPGVG, which is known to be a key component in elastin and exhibits interesting self-assembly behavior (random coil to helix) above its transition temperature. The structure and function of elastin is maintained as long as the glycine and proline residues are present; however, the second valine residue is permissive for any amino acid except proline and is therefore also permissive to ncAAs^20^. Previously, ncAAs have been introduced into ELPs by substituting natural amino acids with structurally similar ncAAs in CFPS systems^49^. Conticello and colleagues^50^ have also previously produced imperfect ELPs containing up to 22 ncAAs in vivo using an *E*. *coli* strain with an attenuated activity of RF1. We previously incorporated up to 30 ncAAs into ELPs by evolving orthogonal synthetases in vivo with enhanced specificities^20^. In this study, we constructed and tested in CFPS three ELP constructs containing 20, 30, and 40 UAG codons, as well as control proteins with tyrosine codons substituted for UAGs.

Before characterizing ELP yields, we first carried out a series of optimization experiments to enhance CFPS yields of sfGFP with 5 UAG codons, as expression yields for this construct were reduced in our initial studies (Fig. 3b). By testing total and soluble protein yields, we determined that the reduction in yield was a result of loss in sfGFP solubility and activity (Supplementary Fig. 13). However, a 31% increase in sfGFP-5UAG production was observed upon increasing pAcFRS levels 2-fold, pAcF levels 2.5-fold, and o-tz-tRNA^opt^ 3-fold (Supplementary Fig. 14). Upon application of these optimized conditions, called OTS^opt^, to the synthesis of ELP-UAGs containing 20, 30, and 40-mers, total yields increased by 40%, 33%, and 26%, respectively, as compared with supplementing with OTS levels optimized for 1 ncAA incorporation (Supplementary Fig. 15). ELP-UAG products were visualized using an autoradiogram, which demonstrated the high percentage of full-length protein and whose band intensities corroborate total yields measured (Supplementary Fig. 15b).

**Fig. 4:**
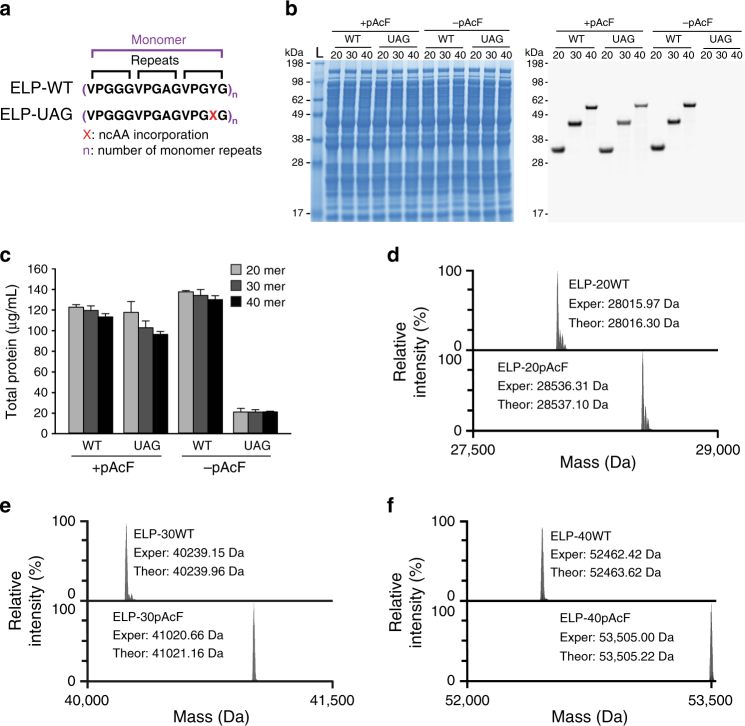
Multi-site ncAA incorporation at high yield and purity. **a** Schematic of the protein sequences for wild-type ELPs containing three pentapeptide repeats per monomer unit (ELP-WT) and ELPs containing 1 ncAA per monomer unit (ELP-UAG). **b** SDS-PAGE and autoradiogram analysis of cell-free produced ELP-WT and ELP-UAG 20-, 30-, and 40-mers in the presence ( + ) and absence ( – ) of *p*-acetyl-L-phenylalanine (pAcF). Numbers next to the molecular weight ladder (L) represent the approximate kilodalton (kDa) size of the band. **c** Total protein yields of cell-free synthesized ELPs (20-, 30, and 40-mers) after incubation at 30 °C for 20 h are shown. Three independent batch CFPS reactions were performed for each sample (*n* = 3). Error bar = 1 SD. **d**–**f** Deconvoluted mass spectra of ELPs obtained by top-down mass spectrometry illustrate complete, site-specific incorporation of pAcF at **d** 20, **e** 30, and **f** 40 sites. Deconvoluted average masses for the major peaks in each spectrum (Exper) match the theoretical average mass (Theor) for each species within 1.2 Da. Smaller peaks next to the major peaks arise from minor oxidation of the protein during electrospray ionization

We next applied OTS^opt^ to the synthesis of ELP-UAGs with 20, 30, and 40-mers in the presence and absence of pAcF to demonstrate specificity of incorporation. ELP-UAGs were only synthesized in the presence of pAcF without any clear indication of truncation products, whereas no protein was observed in the absence of pAcF (Fig. 4). We anticipated that yields would decrease as the number of UAG codons increased due to the higher demand of pAcF-charged o-tRNA. In contrast, near wild type yields of ~ 100 mg/L were obtained for all UAG constructs. We then carefully examined the efficiency of multi-site ncAA incorporation using top-down liquid chromatography (LC)-MS of intact ELPs. LC-MS analysis showed ≥ 98% site-specific pAcF incorporation in ELP-UAG constructs of 20, 30, and 40-mers (Fig. 4).

## Discussion

We present a crude extract-based CFPS platform based on the fully recoded *C321*.*∆A* strain that is capable of high-level protein expression. This platform was generated using MAGE to create libraries of improved extract chassis strains by targeting the functional inactivation of multiple negative effectors. A combinatorial disruption of the genes *endA*^−^, *gor*^−^, *rne*^−^, and *mazF*^−^ (*C321*.*∆A*.*759*) increased total CFPS yields from 397 ± 24 mg/L in the parent strain to 1,780 ± 30 mg/L of sfGFP, which is the highest reported protein yield from RF1-deficient extracts. These improvements translated to the enhanced yield of proteins harboring site specifically introduced ncAAs.

By optimizing the cell-free environment for multiple-identical ncAA incorporation, we were able to achieve multi-site ncAA incorporation into multiple model proteins with high yields (~ 99% wild-type sfGFP expression yields for up to 2 ncAAs, ~ 95% wild-type ELP expression yields for up to 20 ncAAs, and ~ 85% wild-type ELP expression yields for up to 30 and 40 ncAAs) and purity ( ≥ 98% accuracy of ncAA incorporation) due to the absence of RF1 in our system. To our knowledge, these are the purest polymers with this many site-specifically introduced ncAAs (i.e., 40) synthesized to date. This exceeds our previous effort in cells that could synthesize ELP constructs with 30 UAG codons with 71% of the proteins having the desired 30 pAcF residues^20^. As such, our approach opens opportunities to site-specifically modify the dominant physical and biophysical properties of biopolymers. This will allow researchers to go beyond tag-and-modify approaches that have historically been the focus of ncAA incorporation efforts, because the field was previously limited to only one or a few instances of site-specific incorporation. Notably, our protein expression yields of ~ 1,700 mg/L and 99% suppression efficiency for sfGFP with 2 ncAAs outperform the best expression of proteins with single or multiple ncAAs in vivo, to our knowledge (Fig. 2d and Supplementary Fig. 13). For example, previous in vivo experiments using an RF1 knockout strain demonstrated the synthesis of enhanced GFP containing one, two, and three pAcFs at 3.5, 3.5, and 5.4 mg/L, respectively, corresponding to amber suppression efficiencies of 23%, 23%, and 36%^51^. Also in a separate report, an *E*. *coli* strain with attenuated RF1 activity produced 21, 17, and 27 mg/L of sfGFP with 3, ELP with 12, and ELP with 22 ncAAs incorporated, respectively^50^. However, increasing the number of amber codons (i.e., 12 and 22 ncAA incorporation) resulted in numerous truncation products. Here we demonstrate a cell-free system from engineered recoded bacteria that enables high yields of proteins containing up to 40 ncAAs with no observable truncation products. Thus, our cell-free system will serve as a complement to in vivo methods and be a useful technology for developing OTSs for robust synthesis of modified proteins.

Looking forward, incorporating our discovered mutations into a recently published optimized strain, *C321*.*∆A*.*opt*, might further increase the protein expression yields.^52^ In addition, as new genomically recoded organisms with free codons are constructed^24,53–57^, the development of extract chassis strains enabled by our MAGE-guided approach could aid the generation of highly efficient CFPS systems capable of incorporating two or more distinct ncAAs into a single protein or sequence-defined polymer. We envision that the generalized CFPS platform described here will be applied to on-demand biomanufacturing and biomolecular prototyping to transform biochemical engineering and expand the range of genetically encoded chemistry of biological systems.

## Methods

### Strains and plasmids

The bacterial strains and plasmids used in this study are listed in Supplementary Table 3. Details for strain construction, plasmid construction, verification, and culture growth are provided in the Supplementary Methods.

### Cell extract preparation

For prototyping engineered strains, cells were grown in 1 L of 2 × YTPG media (pH 7.2) in a 2.5 L Tunair shake flask and incubated at 34 °C and 220 r.p.m. to OD_600_ of 3.0. Cells were pelleted by centrifuging for 15 min at 5000 × *g* and 4 °C, washed three times with cold S30 buffer (10 mM tris-acetate pH 8.2, 14 mM magnesium acetate, 60 mM potassium acetate, 2 mM dithiothreitol)^58^, and stored at − 80 °C. To make cell extract, the thawed cells were suspended in 0.8 mL of S30 buffer per 1 g of wet cell mass and processed as reported by Kwon and Jewett^59^. Full details for cell growth, collection, and CFPS extract preparation are provided in the Supplementary Methods.

### CFPS reactions

The PANOx-SP system was utilized for CFPS reactions^46^. Briefly, a 15 µL CFPS reaction in a 1.5 mL microcentrifuge tube was prepared by mixing the following components: 1.2 mM ATP; 0.85 mM each of GTP, UTP, and CTP; 34 µg/mL folinic acid; 170 µg/mL of *E*. *coli* tRNA mixture; 13.3 µg/mL plasmid; 16 µg/mL T7 RNA polymerase; 2 mM for each of the 20 standard amino acids; 0.33 mM nicotinamide adenine dinucleotide; 0.27 mM coenzyme-A; 1.5 mM spermidine; 1 mM putrescine; 4 mM sodium oxalate; 130 mM potassium glutamate; 10 mM ammonium glutamate; 12 mM magnesium glutamate; 57 mM HEPES pH 7.2; 33 mM PEP); and 27% v/v of cell extract. For ncAA incorporation, 2 mM pAcF, 0.5 mg/mL pAcFRS, and 10 µg/mL of o-tz-tRNA^opt^ linear DNA were supplemented to cell-free reactions. For multi-site and consecutive ncAA incorporation, OTS^opt^ levels were increased to 5 mM pAcF, 1 mg/mL pAcFRS, and 30 µg/mL o-tz-tRNA^opt^. o-tRNA^opt^ linear DNA was amplified from pY71-T7-tz-o-tRNA^opt^ plasmid as described previously and transcribed during the cell-free reaction^27^. Furthermore, the o-tRNA^opt^ was expressed in the source strain via a plasmid prior to extract preparation. Techniques for purifying aminoacyl tRNA synthetases are described in the Supplementary Methods. When testing the effect of RNAse inhibitor, 1 µL (4U) of inhibitor (Qiagen, Valencia, CA) was added into each 15 µL reaction as per the manufacturer’s suggestion. Each CFPS reaction was incubated for 20 h at 30 °C unless noted otherwise. Fed-batch and scale-up reaction formats are described in the Supplementary Methods.

### Protein quantification

Protein quantification was performed using fluorescence detection and radioactive ^14^C-Leucine incorporation and scintillation counting as described in the Supplementary Methods.

### Whole-genome sequencing

As the gene encoding MutS is inactivated in *C321*.*∆A*, we chose to fully sequence the genomes of six key strains produced during our screening efforts (*C321*.*∆A*, *C321*.*∆A*.*542*, *C321*.*∆A*.*705*, *C321*.*∆A*.*709*, *C321*.*∆A*.*740*, and *C321*.*∆A*.*759*). Whole-genome sequencing was performed by the Yale Center for Genome Analysis for library prep and analysis as described previously^60^ (see Supplementary Methods). Genomes have been deposited to NCBI SRA collection, accession number PRJNA361365 (Individual accession numbers: SRX2511757-SRX2511762).

### Nucleotide and amino acid quantitation using HPLC

Amino acid and nucleotide concentrations were measured via high-performance LC (HPLC). Cell-free reactions were clarified by precipitation with an equal volume 5% w/v trichloroacetic acid. Samples were centrifuged at 12,000 × *g* for 15 min at 4 °C and the supernatant stored at − 80 °C until analyzed using an Agilent 1290 series HPLC system (Agilent, Santa Clara, CA). For amino acid analysis, a Poroshell HPH-C18 column (4.6 × 100 mm, 2.7 µm particle size; Agilent) was used with an automatic pre-column derivatization method using *o-*pthalaldehyde and fluorenylmethyl chloroformate^61^. Nucleotides were analyzed using a BioBasic AX column (4.6 × 150 mm, 5 µm particle size; Thermo Scientific, West Palm Beach, FL). Full methods are described in the Supplementary Methods.

### Full-length sfGFP and ELP purification and MS analysis

To confirm pAcF incorporation at corresponding amber sites, LC-MS analysis was performed on purified sfGFP and ELP reporter protein constructs with pAcF putatively incorporated. MS procedures and sample preparation details are given in the Supplementary Methods.

### Data availability

All data generated or analyzed during this study are included in this published article (and its supplementary files) or are available from the corresponding authors on reasonable request. Genome sequences can be found at: https://www.ncbi.nlm.nih.gov/bioproject/PRJNA361365/. Individual accession codes are: *C321.∆A* (https://www.ncbi.nlm.nih.gov/sra/SRX2511762), *C321.∆A.542* (https://www.ncbi.nlm.nih.gov/sra/SRX2511761), *C321.∆A.705* (https://www.ncbi.nlm.nih.gov/sra/SRX2511760), *C321.∆A.709* (https://www.ncbi.nlm.nih.gov/sra/SRX2511759), *C321.∆A.740* (https://www.ncbi.nlm.nih.gov/sra/SRX2511758), and *C321.∆A.759* (https://www.ncbi.nlm.nih.gov/sra/SRX2511757).

## Electronic supplementary material

Supplementary Information(PDF 9202 kb)
