# Supplementary material for: Cell-free protein synthesis from genomically recoded bacteria enables multisite incorporation of noncanonical amino acids
Source: Nat Commun. 2018 Mar 23;9:1203. doi: 10.1038/s41467-018-03469-5 (PMC5865108; doi:10.1038/s41467-018-03469-5)
Supplement: Supplementary file 1 — Supplementary Information(PDF 9202 kb) [file 41467_2018_3469_MOESM1_ESM.pdf]

Supplementary Figures

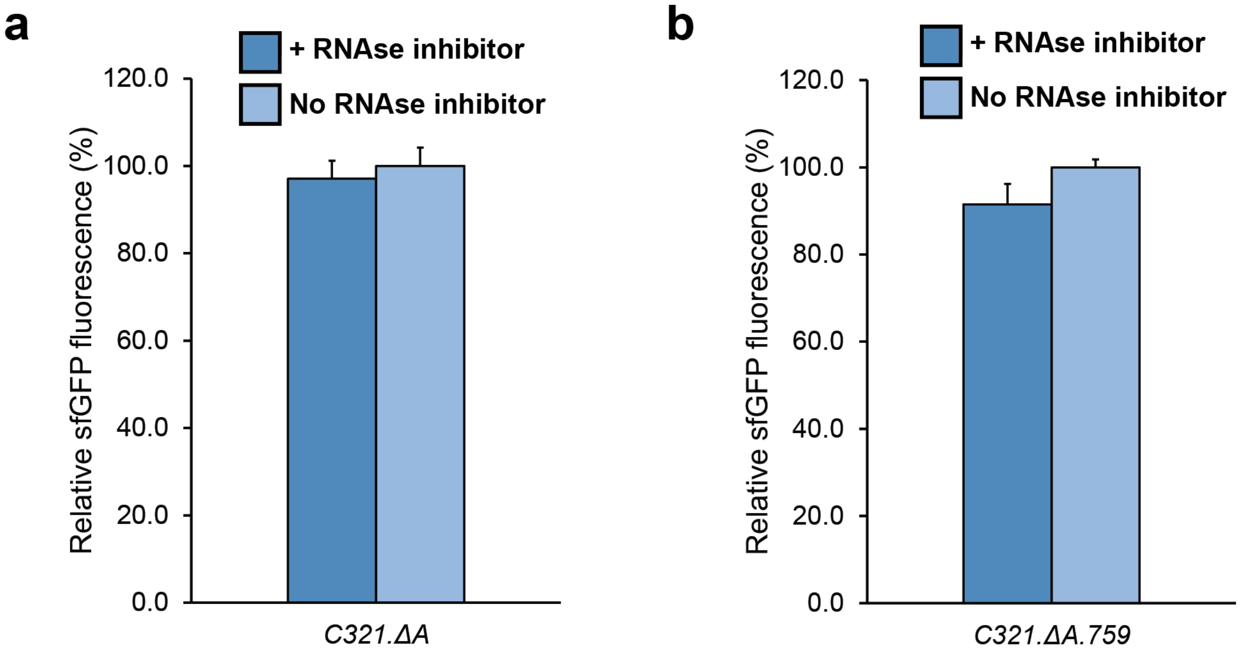

Supplementary Figure 1. Impact of supplementing RNAse inhibitor into CFPS reactions. Cell-free protein synthesis (CFPS) reactions were performed using both *C321.ΔA* **(a)** and *C321.ΔA.759* **(b)** lysates, both with and without supplementation with RNAse inhibitor. Reactions were run for 20 hours at 30 ˚C. For each lysate, relative superfolder green fluorescent protein (sfGFP) fluorescence is shown with the No RNAse inhibitor condition set to 100%. For each condition *n*=3, error bar = 1 standard deviation. These data show that addition of RNAse inhibitor to reactions does not convey the same benefit to CFPS productivity as the functional nuclease knockouts made during the course of engineering strain *C321.ΔA.759*.

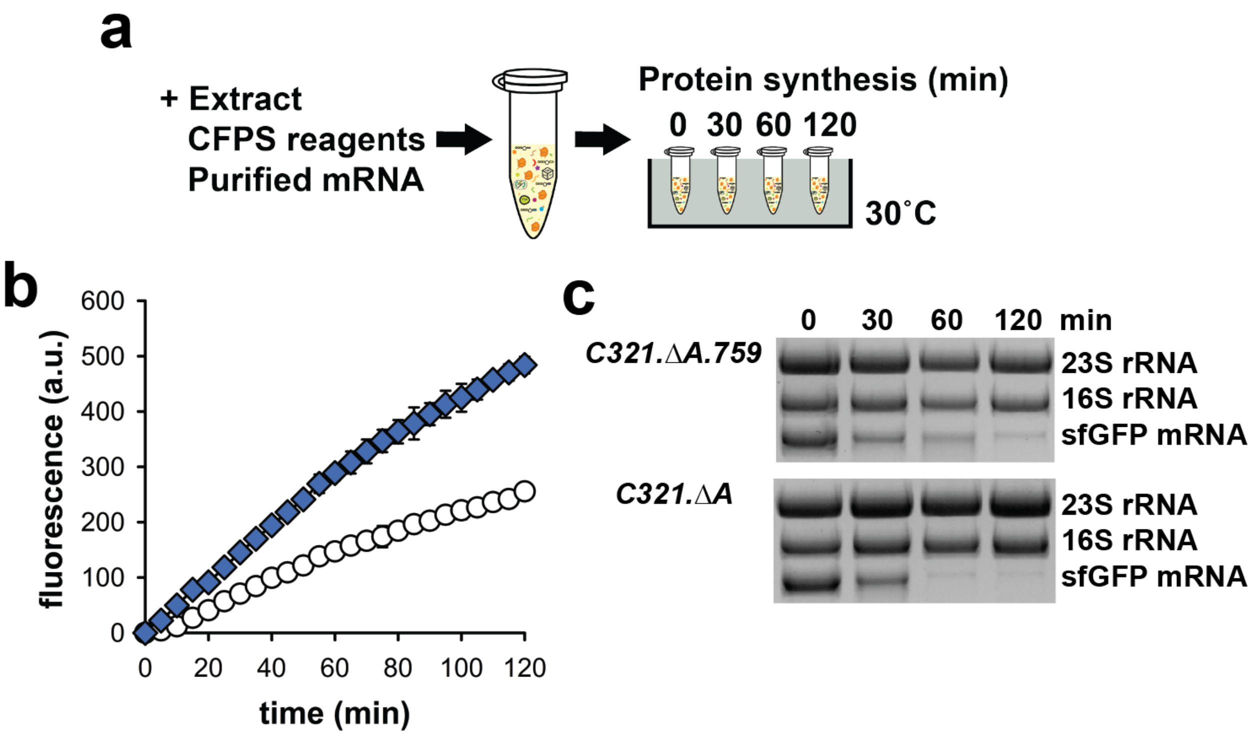

Supplementary Figure 2. mRNA is stabilized in *C321.ΔA.759* lysates. (a) Cell-free translation-only ((TL)-only) reactions synthesizing superfolder green fluorescent protein (sfGFP) were performed in *C321.ΔA.759* and *C321.ΔA* extracts using purified mRNA as template. Reactions were performed for 120 min at 30 ˚C. Throughout the reactions, sfGFP synthesis was monitored by fluorescence **(b)**, and mRNA levels were assessed by an RNA gel **(c)** and analyzed using densitometry. For the mRNA gels, 23S rRNA (2904 nucleotides) and 16S rRNA (1541 nucleotides) are shown as loading and size reference controls for each lane. The sfGFP mRNA with the promoter, gene sequence, and terminator is 917 nucleotides. For each condition *n*=3, error bar = 1 standard deviation. These data show that sfGFP mRNA added to reactions using *C321.ΔA.759* lysate persists longer than in reactions using *C321.ΔA* lysates. This increased mRNA stability likely contributes to the increase in productivity observed here for *C321.ΔA.759* lysates relative to *C321.ΔA* lysates in cell-free protein synthesis.

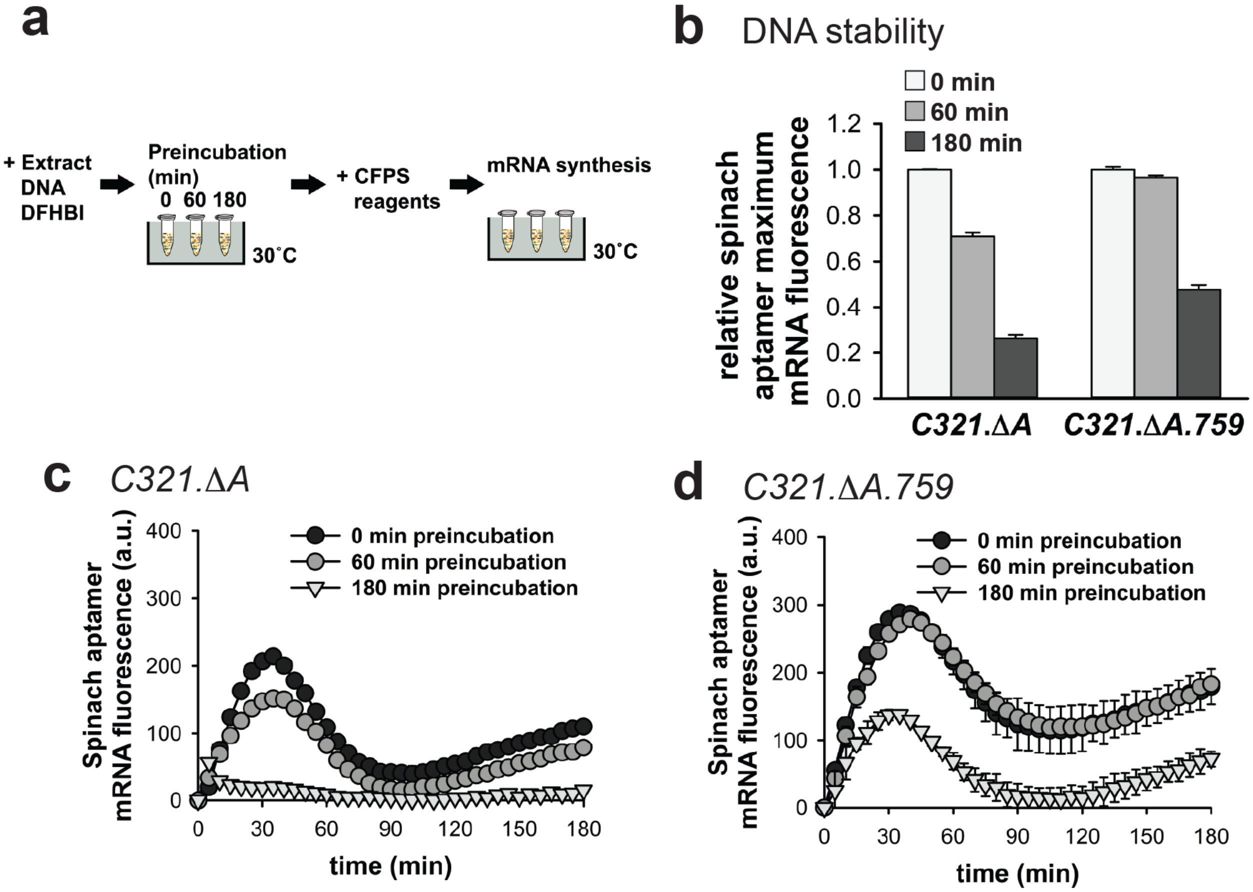

Supplementary Figure 3. DNA is stabilized in *C321.ΔA.759* lysates. (a) To assess template plasmid DNA stability in recoded strain lysates, plasmid encoding the RNA Spinach aptamer was preincubated in lysates. During this preincubation, template DNA is subjected to the activities of any DNAses present in each lysate. After preincubation for 0, 60, or 180 min, CFPS reagents were added and the complete reactions were incubated at 30˚C. High DNAse activity would be expected to catalyze template degradation during preincubation, resulting in a decreased capacity of the complete reaction to synthesize product. DFHBI = 3,5-difluoro-4-hydroxybenzylidene imidazolinone. **(b)** Endpoint measurements of relative Spinach aptamer fluorescence using *C321.ΔA.759* and *C321.ΔA* lysates after preincubation of template plasmid DNA in each lysate for 0, 60, and 180 minutes. **(c and d)** Relative mRNA synthesis level time course experiments from transcription-only (TX-only) reactions using endonuclease I-present (*C321.ΔA*) **(c)** and endonuclease I-deficient (*C321.ΔA.759*) **(d)** extract are compared. While panel **b** captures the relative maximum mRNA level, panels **c** and **d** capture the data measured at 5-minute intervals over a three-hour reaction. For each condition n=3, error bar = 1 standard deviation. Collectively these data show that Spinach aptamer synthesis is increased in transcription-only reactions using *C321.ΔA.759* lysates relative to *C321.ΔA* lysates when template DNA is preincubated in each lysate prior to reaction assembly. This result suggests that template DNA stability is improved in lysates derived from the endonuclease I-deficient strain as a result of reduced DNAse activity.

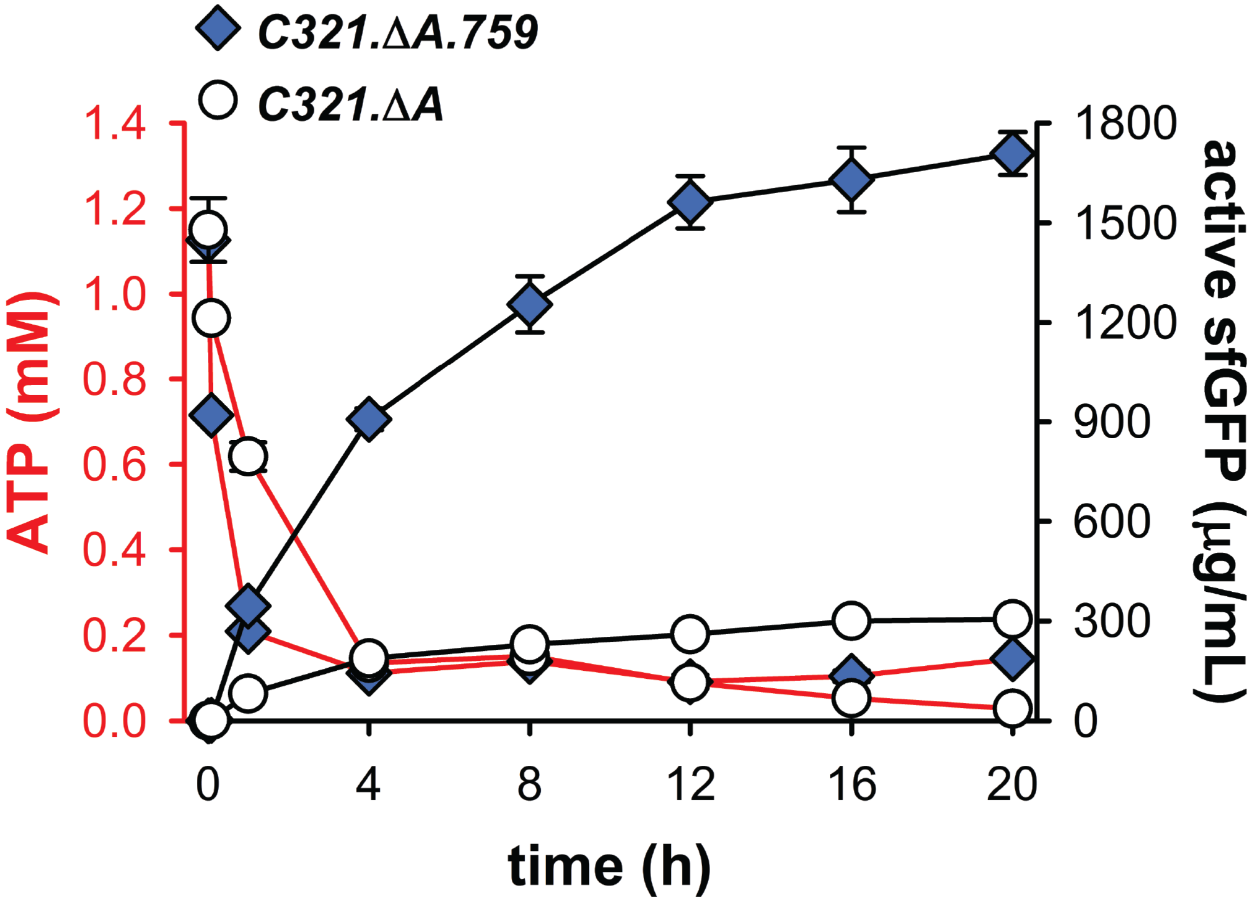

Supplementary Figure 4. Energy stability and CFPS yields in recoded strain lysates. Active superfolder green fluorescent protein (sfGFP) and adenosine triphosphate (ATP) were quantified every 4 h for 20 h in cell-free protein synthesis (CFPS) reactions using *C321.ΔA.759* or *C321.ΔA* extracts. For each condition *n*=3, error bar = 1 standard deviation.

a

$$\text{energy charge} = \frac{[\text{ATP}] + {}^{1}/_{2}[\text{ADP}]}{[\text{ATP}] + [\text{ADP}] + [\text{AMP}]}$$

b

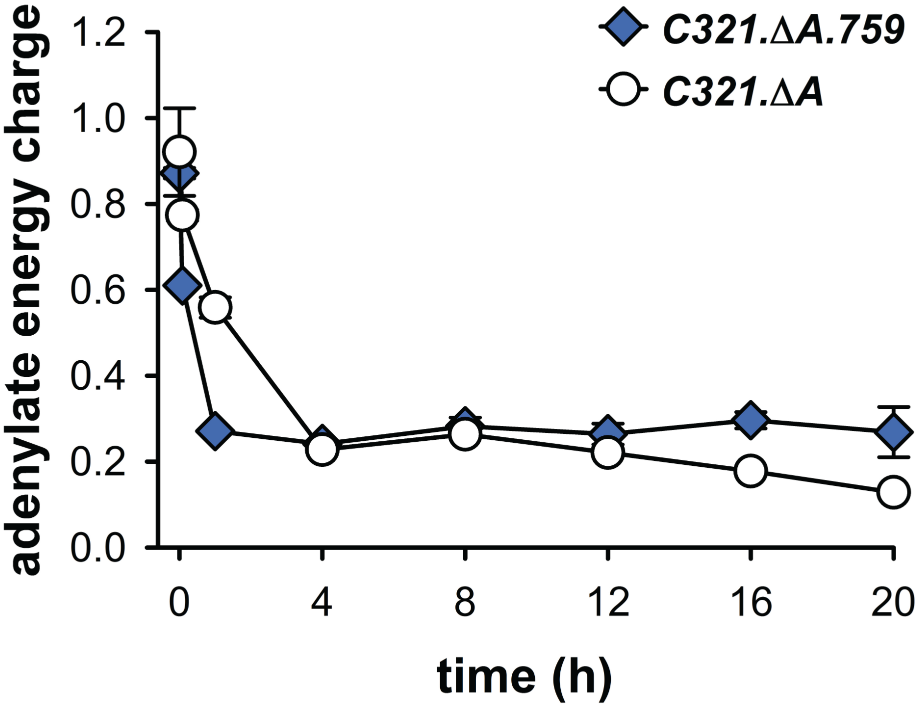

Supplementary Figure 5. Adenylate energy charge of *C321.ΔA.759* and *C321.ΔA*. (a) The formula for calculating adenylate energy charge as described by Atkinson[1]. Energy charge for cell-free protein synthesis (CFPS) reactions using either *C321.ΔA.759* or *C321.ΔA* extracts were determined using this equation. **(b)** Energy charge plotted as a function of reaction time for CFPS reactions using extracts derived from either *C321.ΔA.759* or *C321.ΔA*. For each condition n=3, error bar = 1 standard deviation.

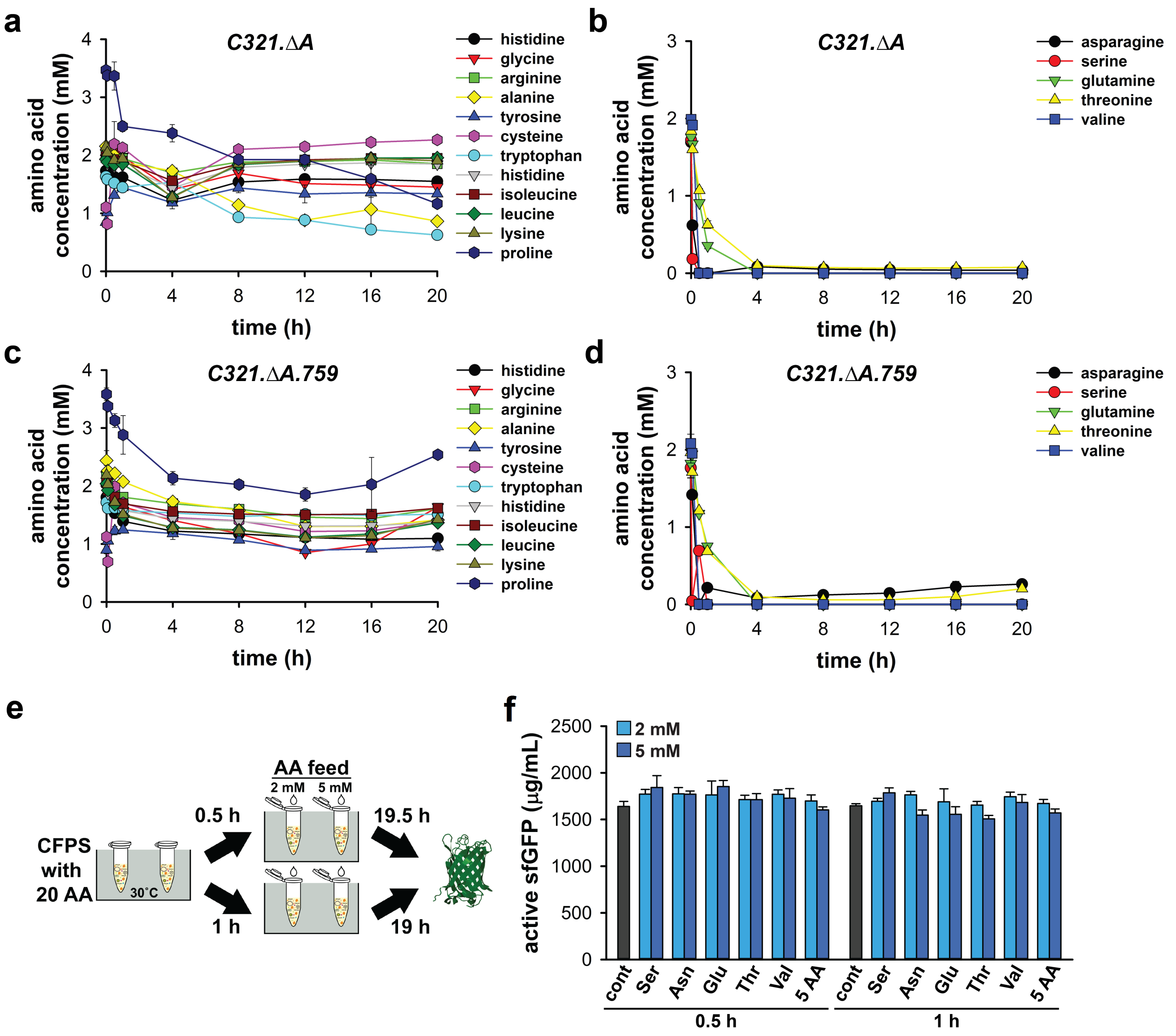

Supplementary Figure 6. Feeding of amino acid substrates into CFPS reactions. Concentrations of amino acid species in cell-free protein synthesis (CFPS) reactions performed using *C321.ΔA* **(a and b)** and C*321.ΔA.759* **(c and d)** extracts were quantified every 4 h for a total reaction time of 20 h. Amino acids found to be ≥90% depleted after 4 hours of incubation **(b and d)** were deemed potentially limiting amino acids. **(e)** To see if amino acid availability was limiting CFPS productivity, reactions were fed with individual potentially limiting amino acids and an equimolar mixture (5 AA) at concentrations of 2 and 5 mM at 0.5 and 1 h time points using 0.5 μL of feeding solution; water was used as a control. **(f)** Endpoint superfolder green fluorescent protein (sfGFP) yields from reactions fed potentially limiting amino acids. For each condition *n*=3, error bar = 1 standard deviation.

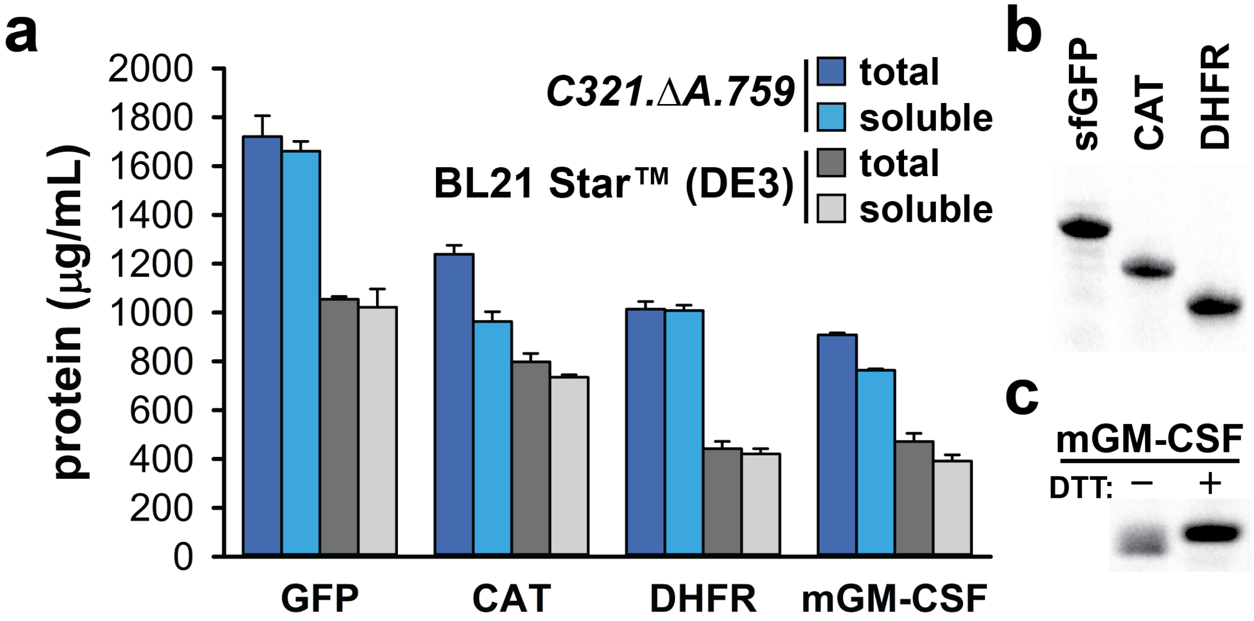

Supplementary Figure 7. *C321.ΔA.759* lysates are versatile and highly-productive. To demonstrate general protein synthesis capabilities, *C321.ΔA.759* lysates were directed in cell-free protein synthesis (CFPS) towards the production of superfolder green fluorescent protein (sfGFP; 26.8 kDa), chloramphenicol acetyltransferase (CAT; 27.7 kDa), dihydrofolate reductase (DHFR; 17.9 kDa), and murine granulocyte-macrophage colony-stimulating factor (mGM-CSF; 16.5 kDa). Reactions were carried out for 20 h at 30 ˚C. **(a)** Total and soluble endpoint yields of the listed proteins synthesized in CFPS using *C321.ΔA.759* lysates. For comparison, yields of the same proteins synthesized using lysates derived from BL21 Star (DE3) are shown. For each condition *n*=3, error bar = 1 standard deviation. **(b)** Autoradiogram showing fully reduced and denatured sfGFP, CAT, and DHFR produced using *C321.ΔA.759* extract in CFPS. **(c)** Autoradiogram of mGM-CSF as produced by *C321.ΔA.759* in CFPS under oxidizing conditions. mGM-CSF was analyzed under oxidizing (-DTT) and reducing (+DTT) conditions. The difference in band position is influenced by the presence of disulfide bonds. These results demonstrate that *C321.ΔA.759* lysates are versatile, capable of successfully synthesizing a variety of proteins varying in both size and structural complexity. Furthermore, these results highlight the high productivity of the engineered lysates, which were consistently able to outperform lysates derived from commercial strain BL21 Star (DE3).

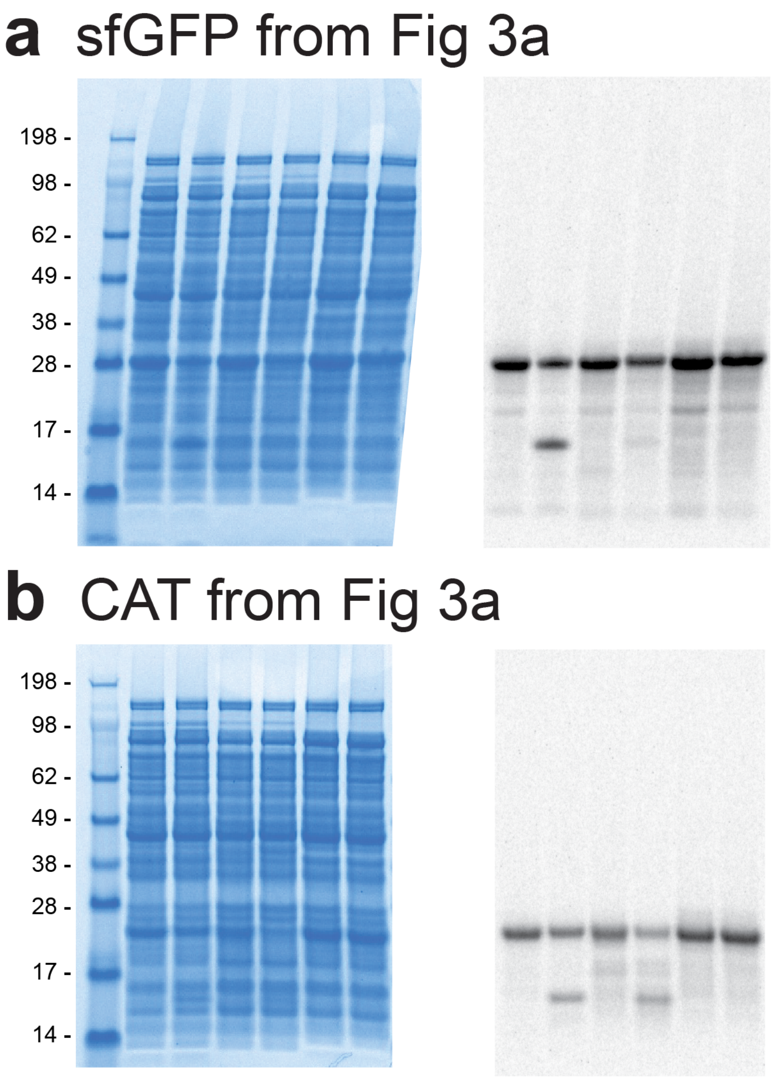

Supplementary Figure 8. Full images of results shown in Fig 3a of the main text. Complete protein gel and autoradiogram images for amber mutants of superfolder green fluorescent protein (sfGFP) and chloramphenicol acetyltransferase (CAT) synthesized using various lysates. A section of each autoradiogram is included in panel 3a of the main text. Numbers to the left of the molecular weight ladder represent the approximate kilodalton size of the band. Molecular weight of sfGFP is ~27 kDa. Molecular weight of CAT is ~28 kDa.

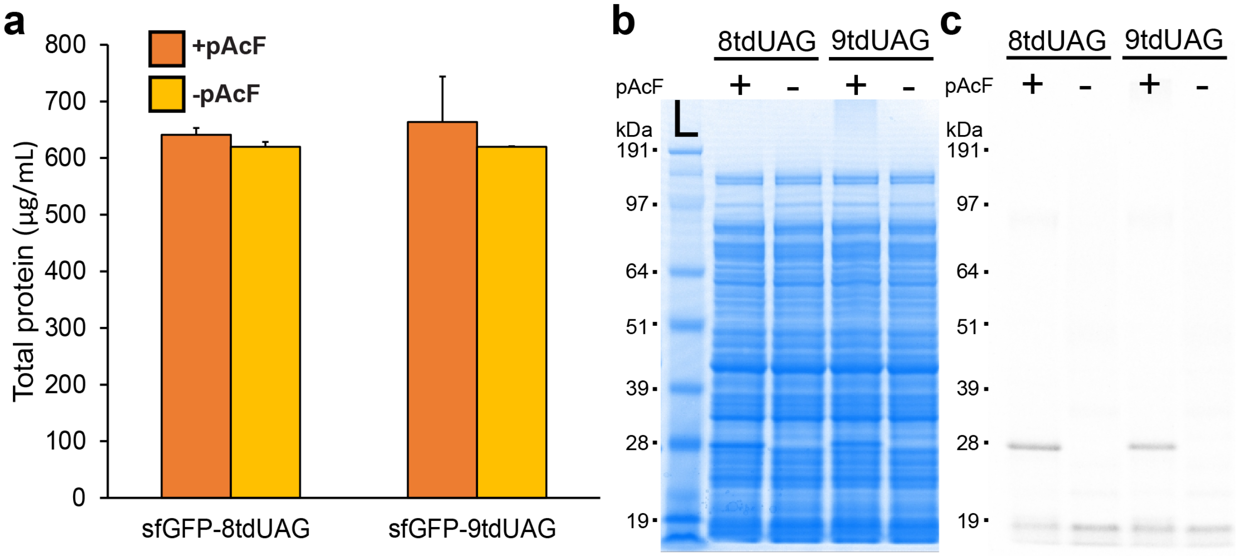

Supplementary Figure 9. Incorporation of consecutive non-canonical amino acids. (a) Shown are total yields of superfolder green fluorescent protein (sfGFP) variants with 8 consecutive amber codons (8tdUAG) or 9 consecutive amber codons (9tdUAG) synthesized in cell-free protein synthesis (CFPS) using lysates derived from *C321.ΔA.759* harboring pEVOL-pAcF. Reactions were performed both in the presence and absence of the non-canonical amino acid (ncAA) *p*-acetyl-L-phenylalanine (pAcF), and were incubated at 30 °C for 20 h. Quantification was achieved via incorporation of ^{14}C-labeled leucine followed by scintillation counting. For each condition *n*=3, error bar = 1 standard deviation. **(b and c)** Protein gel **(b)** and autoradiogram **(c)** analysis of sfGFP-8tdUAG and sfGFP-9tdUAG synthesized in the presence and absence of pAcF using *C321.ΔA.759* lysates. These data show that *C321.ΔA.759* lysates are able to synthesize full-length protein featuring multiple consecutive ncAAs, but only when the ncAA is supplied to the system. While the results of panel **a** suggest that the same concentration of product is synthesized whether or not pAcF is present, the autoradiogram image in panel **c** shows that the protein synthesized in the absence of pAcF is truncated and that full-length protein is only obtained when pAcF is added to reactions.

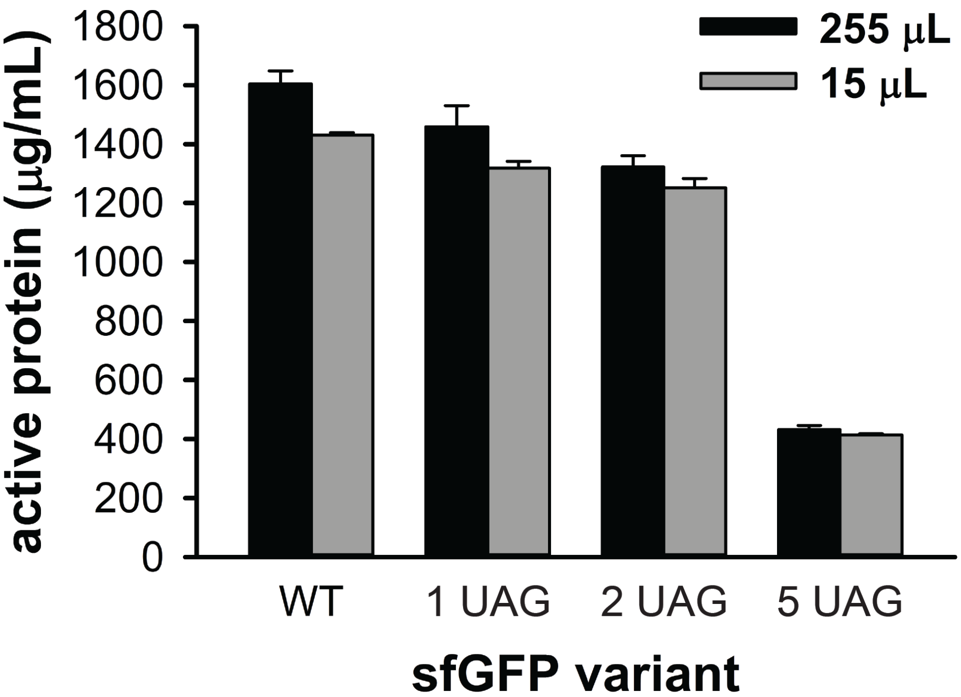

Supplementary Figure 10. Scaled-up synthesis of sfGFP containing multiple ncAAs. Active yields of wild-type superfolder green fluorescent protein (sfGFP) as well as mutant sfGFP featuring 1, 2, and 5 amber codons (1 UAG, 2 UAG, and 5 UAG, respectively) are shown for 15 µL and 255 µL batch mode cell-free protein synthesis (CFPS) reactions using lysates derived from *C321.ΔA.759* harboring pEVOL-pAcF. Amber codons were suppressed with the non-canonical amino acid (ncAA) *p*-acetyl-L-phenylalanine (pAcF). Reactions were performed at 30 ˚C for 20 h. For each condition n=3, error bar = 1 standard deviation. These data show that CFPS using *C321.ΔA.759* lysates can be scaled up 17-fold with no loss in productivity.

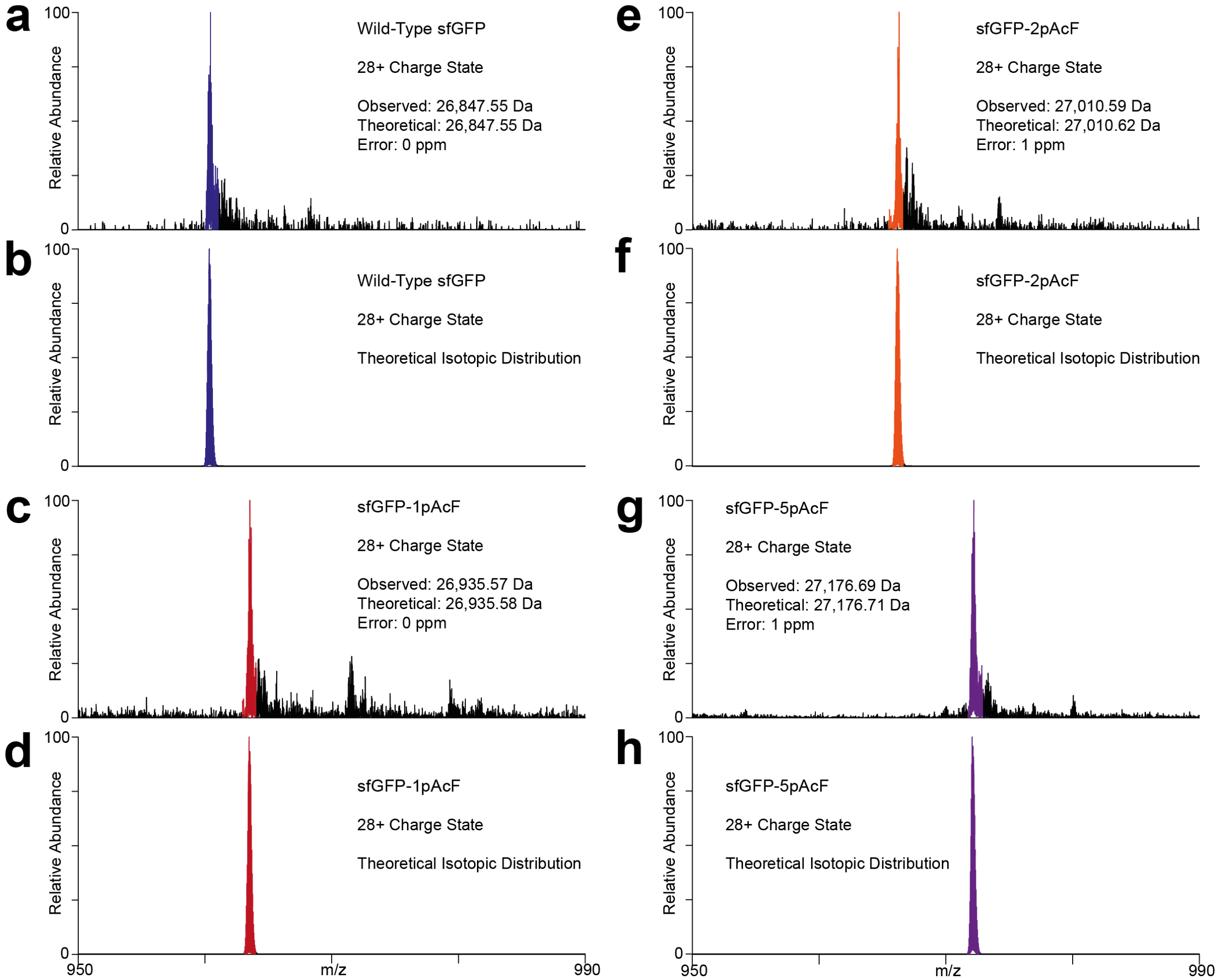

Supplementary Figure 11. Mass spectrometry analysis of pAcF incorporation into sfGFP. Superfolder green fluorescent protein (sfGFP) variants were synthesized in cell-free protein synthesis (CFPS) reactions using lysates derived from *C321.ΔA.759* harboring pEVOL-pAcF. Following CFPS, top-down mass spectrometry was used to assess the degree of *p*-acetyl-L-phenylalanine (pAcF) incorporation into amber codons for each sfGFP species. **(a and b)** experimental **(a)** and theoretical **(b)** mass peaks for wild-type sfGFP; **(c and d)** experimental **(c)** and theoretical **(d)** mass peaks for sfGFP containing a single pAcF corresponding to the position of T216; **(e and f)** experimental **(e)** and theoretical **(f)** mass peaks for sfGFP containing two pAcFs corresponding to the positions of N212 and T216; **(g and h)** experimental **(g)** and theoretical **(h)** mass peaks for sfGFP containing five pAcFs corresponding to the positions of D36, K101, E132, D190, and E213. Analysis of these data shows that site-specific incorporation of pAcF was ≥98% in all samples, with ≤1 ppm difference between experimental and theoretical protein masses. This shows that *C321.ΔA.759* lysates are capable of efficient and high-yielding site-specific incorporation of pAcF into proteins.

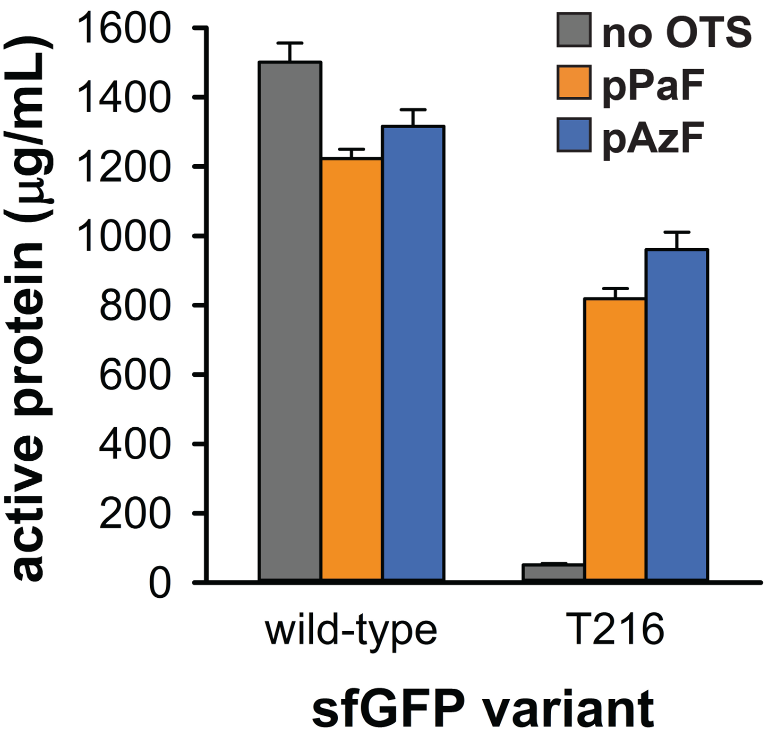

Supplementary Figure 12. *C321.ΔA.759* lysates are compatible with multiple ncAAs. The non-canonical amino acids (ncAAs) *p*-propargyloxy-L-phenylalanine (pPaF) and *p*-azido-L-phenylalanine (pAzF) were incorporated in CFPS into two superfolder green fluorescent protein (sfGFP) variants using extracts derived from *C321.ΔA.759* overexpressing an optimized orthogonal amber suppressor tRNA (o-tRNA) from the pDULE plasmid. Both wild-type sfGFP and an sfGFP variant featuring an amber codon at position T216 were synthesized in the presence of each ncAA's orthogonal translation system (OTS) by supplementing reactions with purified orthogonal synthetase corresponding to either pPaF or pAzF. Yields of active protein for both variants of sfGFP synthesized in the presence of each OTS are shown. For each condition *n*=3, error bar = 1 standard deviation. These data shown that *C321.ΔA.759* is compatible with multiple OTSs, demonstrating generality in the strain's ability to facilitate site-specific incorporation of several different kinds of ncAAs into proteins.

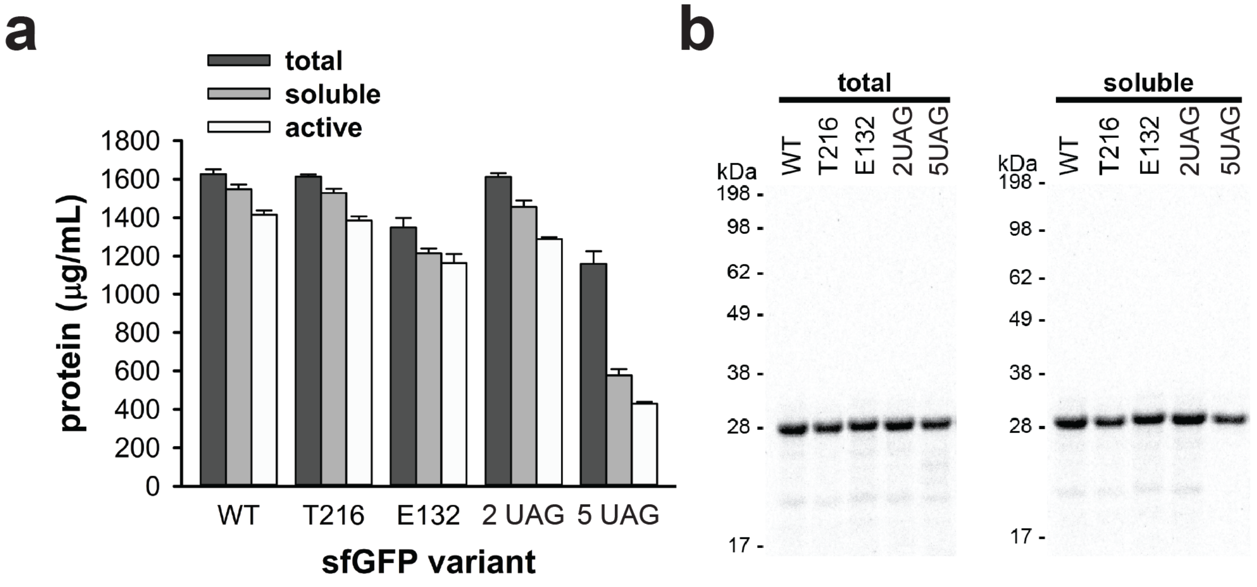

Supplementary Figure 13. Yields for sfGFP containing multiple identical ncAAs. Extract derived from *C321.ΔA.759* expressing orthogonal translation component for *p*-acetyl-L-phenylalanine (pAcF) was used to catalyze cell-free protein synthesis (CFPS) reactions synthesizing superfolder green fluorescent protein (sfGFP) variants. The sfGFP variants used were wild-type (WT), sfGFP containing a single pAcF corresponding to the position of T216 (T216), sfGFP containing a single pAcF corresponding to the position of E132 (E132), sfGFP containing two pAcFs corresponding to the positions of N212 and T216 (2 UAG), and sfGFP containing five pAcFs corresponding to the positions of D36, K101, E132, D190, and E213 (5 UAG). **(a)** Shown are total and soluble yields (measured using radioactive incorporation) and active yields (measured using fluorescence) for each sfGFP variant synthesized. For each condition n=3, error bar = 1 standard deviation. **(b)** Total and soluble protein for each sfGFP variant was visualized via autoradiograms (molecular weight of sfGFP is 27 kDa). Inspection of the data in panel **(a)** reveals that the reduction in yield for the sfGFP-5UAG construct can be mostly attributed to a loss in sfGFP solubility and activity.

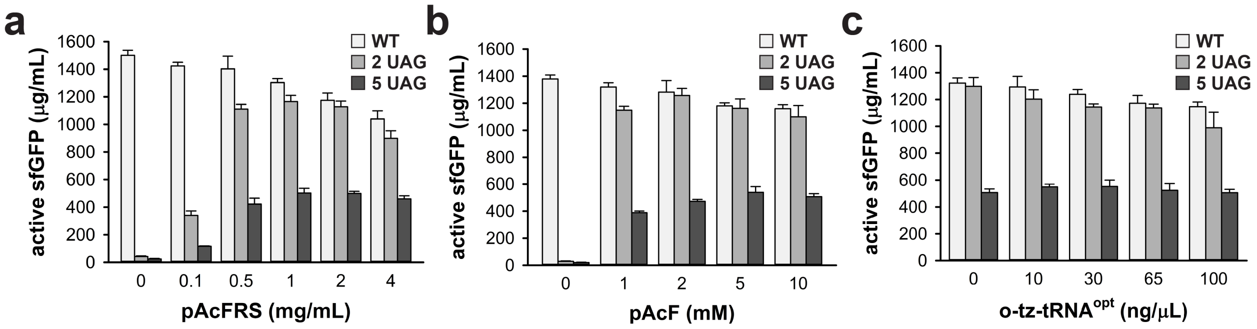

Supplementary Figure 14. Optimization of pAcF incorporation into sfGFP-5UAG. Using extracts derived from *C321.ΔA.759* expressing orthogonal translation components for *p*-acetyl-L-phenylalanine (pAcF), we optimized levels of **(a)** purified orthogonal pAcF-specific synthetase (pAcFRS), **(b)** non-canonical amino acid (pAcF), **(c)** and orthogonal transzyme $tRNA^{opt}$ (o-tz-$tRNA^{opt}$) added into the cell-free protein synthesis (CFPS) reactions. Varying levels of each of the aforementioned components were added to reactions directed to synthesize one of three different forms of superfolder green fluorescent protein (sfGFP), namely wild-type sfGFP, sfGFP containing two pAcFs corresponding to the positions of N212 and T216 (2 UAG), and sfGFP containing five pAcFs corresponding to the positions of D36, K101, E132, D190, and E213 (5 UAG). Yields are shown here. For each condition n=3, error bar = 1 standard deviation. These data show that the synthesis of sfGFP-5UAG is improved when additional pAcF-specific translational components (pAcFRS, pAcF, and o-tz-$tRNA^{opt}$) are added to CFPS reactions.

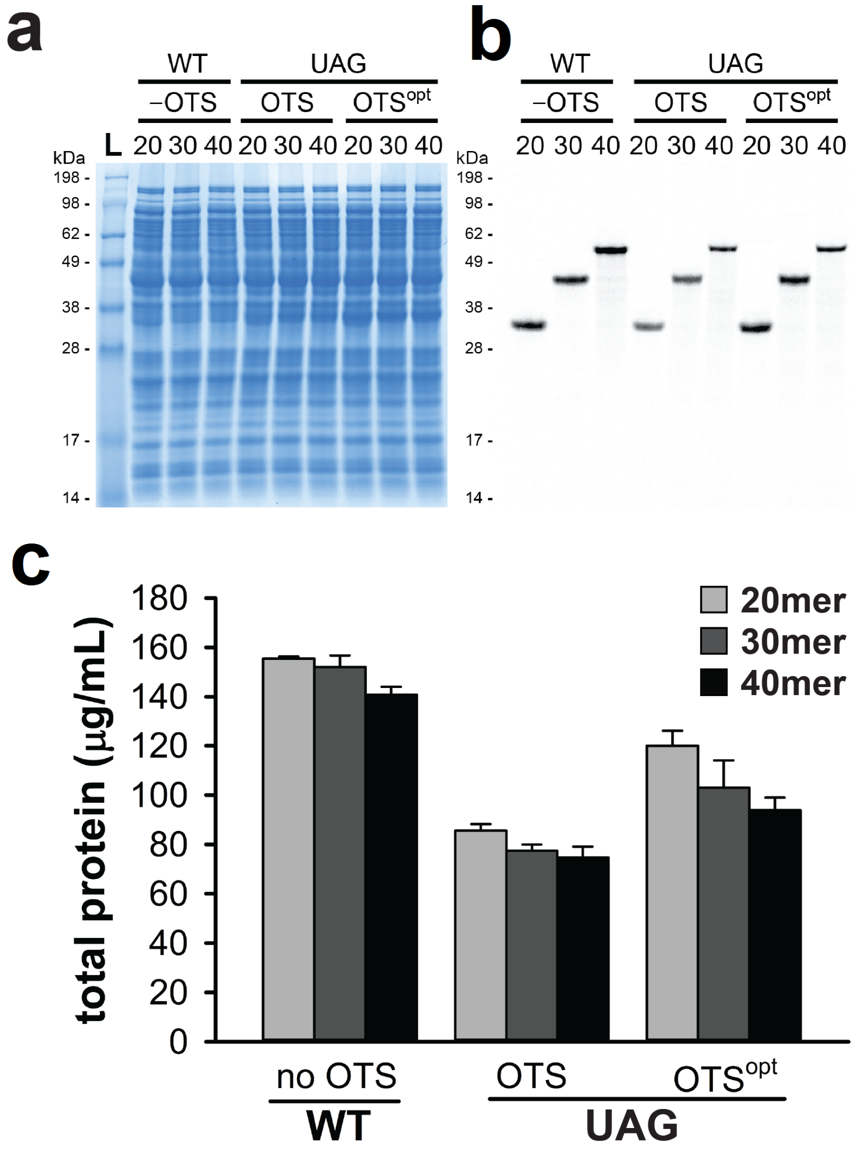

Supplementary Figure 15. Improved full-length ELP-UAG yield using optimized OTS^{opt}. Elastin-like polypeptide (ELP) amber mutant (ELP-UAG) constructs containing 20, 30, and 40 -mers were synthesized in cell-free protein synthesis (CFPS) reactions supplemented with purified orthogonal translation system (OTS) components specific to *p*-acetyl-L-phenylalanine (pAcF). OTS components included pAcF synthetase (pAcFRS), pAcF, and orthogonal transzyme tRNA^{opt} (o-tz-tRNA^{opt}). ELP-UAGs were synthesized under two sets of conditions: OTS^{opt} levels as identified by results from **Supplementary Figure 14** (1 mg/mL pAcFRS, 5 mM pAcF, 30 ng/μL o-tz-tRNA^{opt}), or standard OTS levels (0.5 mg/mL pAcFRS, 2 mM pAcF, 10 ng/μL o-tz-tRNA^{opt}). ELP-WT variants were synthesized in the absence of the OTS as controls. Products were visualized by SDS-PAGE **(a)** an autoradiogram **(b)**, and **(c)** total protein was quantified using ^{14}C-glycine incorporation and scintillation counting. For each condition *n*=3, error bar = 1 standard deviation. These results show that the synthesis of ELP-UAG constructs is increased using OTS^{opt} conditions.

Supplementary Tables

Supplementary Table 1. Negative CFPS effectors functionally inactivated in *C321.ΔA*. A summary of putative negative effectors inactivated in engineered variants of *C321.ΔA*. Individual mutation targets were chosen for their potential to stabilize essential substrates, including DNA, RNA, protein, amino acids, and energy supply.

Function	Gene	Reason for disruption	Reference
**DNA Stability**	*endA*	Stabilize DNA	[2, 3, 4]
**RNA Stability**	*mazF*	Stabilize mRNA	[2, 5]
	rna	Stabilize mRNA	[6]
	rnb	Stabilize mRNA	[2, 7, 8]
	rne	Stabilize mRNA	[9]
**Protein Stability**	*gor*	Decrease reducing power of cell extracts to enhance disulfide bond formation	[7, 10]
	lon	ATP-dependent protease	[11]
	ompT	Outer membrane protease	[11, 12]
**Amino Acid Stability**	*gdhA*	Stabilize glutamate	[13]
	gshA	Stabilize cysteine	[7, 14]
	sdaA	Stabilize serine	[15]
	sdaB	Stabilize serine	[15]
	speA	Stabilize arginine	[15]
	tnaA	Stabilize tryptophan	[15]
**Energy Supply**	*glpK*	Conserve ATP	[16, 17]

Supplementary Table 2. DNA sequences used throughout this study. Presented are sequences for primers and oligos used in MAGE, MASC PCR, DNA sequencing, and ELP construction. Also listed are sequences for gblock DNA fragments used in ELP construction. Underlined bold text indicates location of mismatch and insertion of premature stop codon. The first four bases of the 5'-MAGE oligonucleotides were phosphorothioated (*). /Phos/ indicates 5' phosphorylation.

Primer Name	DNA Sequence (listed 5' to 3')
**MAGE**	
rna	G*C*T*G*ATTTCTGACCGTACATGGTCTGTGGCCAGGA**ATGTAAATGCTGATGTA**TTGCCTAAATCGGTTGCTGCCCGTGGTGTTGATGAAC
rne	C*T*G*T*TGAGCCGCTTCTTCGGCGCACTGAAAGCGCTGTTCAGC**TAACTGA**GAAGAAACCAAACCGACCGAGCAACCAGCACCGAAAGCAGA
rnb	A*T*T*T*TGTCACCATCGACAGTGCCAGCACAGAAGATATGGAT**TAACTGA**CTTTTCGCTAAGGCGTTGCCGGATGACAAACTTCAGCTGAT
mazF	T*T*G*A*TTGCGTTGTACAAGGAACACACAGACACATACCTGTTTTGTTGTT**TCAGTTA**GAAAGGACTCAGGACAACAGCTGGACGATGTCC
endA	C*G*G*T*AAAAGTCCACGCTGACGCGCCCGGTACGTTTTATTGC**TAACTGA**AAAATTAACTGGCAGGGCAAAAAAGGCGTTGTTGATCTGCA
ompT	T*G*G*A*CAACTCTCGGCAGCCGAGGTGGCAATATGGTCG**CG**CAGGACTGGATGGATTCCAGTAACCCCGGAACCTGGACGGATGAAAGTAGA
lon	A*A*C*T*CTGCTTCCGCTTTCTCTTTTGCCTCTTTCGGCATCTT**TCAGTTA**GTCGATTTTGCGCTTCAGGGCTTCGTTTTCGTCCGGCGCGT
gor	G*T*T*T*TGATACCACTATCAATAAATTCAACTGGGAAACGTTG**TAACTGA**AGCCGTACCGCCTATATCGACCGTATTCATACTTCCTATGA
gdhA	T*G*C*G*CTTCCATCCGTCAGTTAACCTTTCCATTCTCAAATTC**TAACTGA**TTTGAACAAACCTTCAAAAATGCCCTGACTACTCTGCCGA
sdaA	T*C*T*A*CAGCAAAACTTATTATTCCATCGGCGGCGGTTTTATC**TAACTGA**GAAGAACACTTTGGTCAGGATGCTGCCAACGAAGTAAGCGT
sdaB	A*C*A*G*CCAGACTTACTACTCTATTGGCGGTGGCTTTATCGTT**TAACTGA**GAGCATTTTGGCCAGCAGGATAGCGCACCGGTTGAAGTTCC
speA	A*T*C*T*TCTCAATGACCAGATAGACCTTGTGCCCCATCTTCTC**TCAGTTA**TAATGCCAGGCGGATATATTCGCGGTCTTTATAACCGTTGC
gshA	G*A*T*G*CACCAAACAGATAAGGAATGACCCAACCGAAACGATA**TCAGTTA**GCGGATAACGCGGAAATAGCCCGCAGAAATTTTCTCTTTGG
tnaA	A*C*C*T*TGAGGGATTAGAACGCGGTATTGAAGAAGTTGGTCCG**TAACTGA**GTGCCGTATATCGTTGCAACCATCACCAGTAACTCTGCAGG
glpK	G*C*A*C*CAAAGTGAAGTGGATCCTCGACCATGTGGAAGGCTCT**TAACTGA**CGTGCACGTCGTGGTGAATTGCTGTTTGGTACGGTTGATAC
**MASC PCR**	
rna-wt-F	GTACATGGTCTGTGGCCAGGATTGC
rna-mut-F	GGCCAGGA**ATGTAAATGCTGATGTA**
rna-R	TGGCATGACTTCACTTAGTTTAGC
rne-wt-F	CACTGAAAGCGCTGTTCAGCGGTGGT
rne-mut-F	CACTGAAAGCGCTGTTCAGC**TAACTGA**
rne-R	GTGCGACTACCGCTTCTTCGGCTAC
rnb-wt-F	CCAGCACAGAAGATATGGATGACGCC
rnb-mut-F	CCAGCACAGAAGATATGGAT**TAACTGA**

rnb-R	TCACTTTCAGGCTGCCAGTCACCGG
mazF-wt-F	CTGTTGTCCTGAGTCCTTTCATGTAC
mazF-mut-F	CTGTTGTCCTGAGTCCTTTC**TAACTGA**
mazF-R	GGCTTTAATGAGTTGTAATTCCTCTG
endA-wt-F	CCCGGTACGTTTTATTGCGGATGT
endA-mut-F	CCCGGTACGTTTTATTGC**TAACTGA**
endA-R	GCTGGCGCTGGTAATTTCGGCGTCA
ompT-wt-F	CAGCCGAGGTGGCAATATGGTCGAT
ompT-mut-F	CAGCCGAGGTGGCAATATGGTCG**CG**
ompT-R	GAGTTCAAAATCTTCATAACGATAAC
lon-wt-F	CTGAAGCGCAAAATCGACGCGGCG
lon-mut-F	CTGAAGCGCAAAATCGAC**TAACTGA**
lon-R	AGCGGGTTTTTCACGCCCACTTTCGC
gor-wt-F	TTCAACTGGGAAACGTTGATCGCC
gor-mut-F	TTCAACTGGGAAACGTTG**TAACTGA**
gor-R	TGCAACATTTCGTCCATACCAAAGC
gdhA-wt-F	CCTTTCCATTCTCAAATTCCTCGGC
gdhA-mut-F	CCTTTCCATTCTCAAATTC**TAACTGA**
gdhA-R	CTGCCGCCAAATGAAAGGCCCTTAC
sdaA-wt-F	ATCGGCGGCGGTTTTATCGTCGAT
sdaA-mut-F	ATCGGCGGCGGTTTTATC**TAACTGA**
sdaA-R	CAAGACCCGCAGCAGCCATTGAACAG
sdaB-wt-F	GGCGGTGGCTTTATCGTTGATGAA
sdaB-mut-F	GGCGGTGGCTTTATCGTT**TAACTGA**
sdaB-R	TACCTGTCCGGCGACCGGGTCACAC
speA-wt-F	GAATATATCCGCCTGGCATTAATTGGC
speA-mut-F	GAATATATCCGCCTGGCATTA**TAACTGA**
speA-R	CGGTGATTACCGTCGGATGCGGCAG
gshA-wt-F	TATTTCCGCGTTATCCGCAATTAC
gshA-mut-F	TATTTCCGCGTTATCCGC**TAACTGA**
gshA-R	AAATCCTCTTCGCGCAGAATTTCCAGC
tnaA-wt-F	GGTATTGAAGAAGTTGGTCCGAATAAC
tnaA-mut-F	GGTATTGAAGAAGTTGGTCCG**TAACTGA**
tnaA-R	CTACCGCCAGACGCTCCATCGCGCC
glpK-wt-F	GACCATGTGGAAGGCTCTCGCGAG
glpK-mut-F	GACCATGTGGAAGGCTCT**TAACTGA**
glpK-R	CAAACAGCGCGGCCTGCTGGTCACC
**DNA Sequencing**	
rna-seq-F	GTTTCTCTGCTTCCCTTCTCTTCT
rne-seq-F	CAGATGGAAACCCCGCACTACCACG

rnb-seq-F	CTGAAAGGCGATCGTTCTTTCTATG
mazF-seq-F	GTAAAGAGCCCGTATTTACGCTTGC
endA-seq-F	ATGTACCGTTATTTGTCTATTGCTGC
ompT-seq-F	CTGACAACCCCTATTGCGATCAGCTC
lon-seq-F	GTGCTGGTGCGTACTGCAATCAGCC
gor-seq-F	TAAACACTATGATTACATCGCCATC
gdhA-seq-F	GCAAGCCGTTCGTGAAGTAATGACC
sdaA-seq-F	TACTCGCGTTGCCGTGGACGTTTATG
sdaB-seq-F	TGACCCGCGTGGTGGTTGACGTGTAC
speA-seq-F	GTGAAAACTCGTGAAGCACAGGGCC
gshA-seq-F	GAACATATGCTGACCTTTATGCGCG
tnaA-seq-F	CGTAGCTACTATGCGTTAGCCGAG
glpK-seq-F	AGGTTGGGTAGAACACGACCCAATG
**tdUAG cloning**	
8tdUAG-E132-f	/Phos/**TAGTAGTAGTAGTAGTAGTAGTAG**GATGGCAATATCCTGGGCC ATAAACTG
9tdUAG-E132-f	/Phos/**TAGTAGTAGTAGTAGTAGTAGTAGTAG**GATGGCAATATCCTGG GCCATAAACTG
tdUAG-E132-r	/Phos/TTTAAAATCCGTGCCTTTCAGTTCAATGCG
**ELP cloning**	
TS-BlpI-R	TAGTTATTGCTCAGCGGTGG
TS-ApaI-F	GTCCCGGGTTATGGGCCC
KpnIApaI-f	GGTCGCGGGGTTGGGCCCAGCAAAGGTGAAGAACTGTTTACCG
KpnIApaI-r	TTTGCTCATGGTACCATCTCCTTCTTAAAGTTAAACAAAATTATTTC
**gBlock DNA sequence**	
gB-Twin-Strep-ApaIBlpI	GTCCCGGGTTATGGGCCCTCGGCGTGGAGCCACCCGCAGTTCGAGAAA GGTGGAGGTTCCGGAGGTGGATCGGGAGGTTCGGCGTGGAGCCACCCG CAGTTCGAAAAATAATAAGTCGACCGGCTGCTAACAAAGCCCGAAAGG AAGCTGAGTTGGCTGCTGCCACCGCTGAGCAATAACTA<br>ApaI, BlpI

Supplementary Table 3. Strains and plasmids used in this study. 'Δ' indicates deleted gene, and superscript '-' indicates disabled/functionally inactivated gene via MAGE. Km^R, Ap^R, Zeo^R and Cm^R are kanamycin, ampicillin, zeocin, and chloramphenicol resistance, respectively.

Strains and plasmids	Genotype/relevant characteristics	Source
**Strains**		
EcNR2	*MG1655 with λ-prophage::bioA/bioB* and *cmR::mutS*	[18]
BL21 (DE3)	*fhuA2 [lon] ompT gal (λ DE3) [dcm] ΔhsdS λ DE3 = λ sBamHIo ΔEcoRI-B int::(lacI::PlacUV5::T7 gene1) i21 Δnin5*	New England Biolabs
BL21 Star (DE3)	F^- *ompT* $hsdS_B$ ($r_B^- m_B^-$) *gal dcm rne131* (DE3)	Life Technologies
MCJ.559	*C13.ΔA. endA⁻ csdA⁻* (C13: 13 TAG recoded to TAA)	[2]
C321.ΔA	Δ*prfA* Ω Cb^R, Zeo^R, EcNR2 derivative with all 321 TAG stop codons reassigned to TAA	[19]
C321.ΔA.540	*C321.ΔA. rnb⁻*	This study
C321.ΔA.541	*C321.ΔA. mazF⁻*	This study
C321.ΔA.542	*C321.ΔA. endA⁻*	This study
C321.ΔA.598	*C321.ΔA. rna⁻*	This study
C321.ΔA.618	*C321.ΔA. ompT⁻*	This study
C321.ΔA.620	*C321.ΔA. glpK⁻*	This study
C321.ΔA.626	*C321.ΔA. gshA⁻*	This study
C321.ΔA.628	*C321.ΔA. tnaA⁻*	This study
C321.ΔA.644	*C321.ΔA. gdhA⁻*	This study
C321.ΔA.666	*C321.ΔA. gor⁻*	This study
C321.ΔA.667	*C321.ΔA. lon⁻*	This study
C321.ΔA.668	*C321.ΔA. rne⁻*	This study
C321.ΔA.669	*C321.ΔA. sdaA⁻*	This study
C321.ΔA.672	*C321.ΔA. sdaB⁻*	This study
C321.ΔA.674	*C321.ΔA. speA⁻*	This study
C321.ΔA.544	*C321.ΔA. endA⁻ mazF⁻*	This study
C321.ΔA.678	*C321.ΔA. endA⁻ glpK⁻*	This study
C321.ΔA.679	*C321.ΔA. endA⁻ tnaA⁻*	This study
C321.ΔA.709	*C321.ΔA. endA⁻ gor⁻*	This study
C321.ΔA.711	*C321.ΔA. endA⁻ rne⁻*	This study
C321.ΔA.708	*C321.ΔA. endA⁻ lon⁻*	This study
C321.ΔA.703	*C321.ΔA. endA⁻ gor⁻ glpK⁻*	This study
C321.ΔA.705	*C321.ΔA. endA⁻ gor⁻ rne⁻*	This study
C321.ΔA.706	*C321.ΔA. endA⁻ gor⁻ tnaA⁻*	This study
C321.ΔA.740	*C321.ΔA. endA⁻ gor⁻ mazF⁻*	This study
C321.ΔA.738	*C321.ΔA. endA⁻ gor⁻ lon⁻*	This study
C321.ΔA.759	*C321.ΔA. endA⁻ gor⁻ rne⁻ mazF⁻*	This study

C321.ΔA.619	*C321.ΔA.* $glpK^-$ $ompT^-$	This study
C321.ΔA.617	*C321.ΔA.* $glpK^-$ rne^-	This study
C321.ΔA.621	*C321.ΔA.* $ompT^-$ rne^-	This study
C321.ΔA.680	*C321.ΔA.* $endA^-$ $glpK^-$ $tnaA^-$	This study
C321.ΔA.664	*C321.ΔA.* $endA^-$ lon^- rna^-	This study
C321.ΔA.756	*C321.ΔA.* $endA^-$ $glpK^-$ gor^- $mazF^-$	This study
C321.ΔA.755	*C321.ΔA.* $endA^-$ gor^- lon^- $mazF^-$	This study
C321.ΔA.758	*C321.ΔA.* $endA^-$ gor^- $mazF^-$ $tnaA^-$	This study
C321.ΔA.879	*C321.ΔA.* $endA^-$ gor^- rna^- rne^-	This study
C321.ΔA.878	*C321.ΔA.* $endA^-$ gor^- rnb^- rne^-	This study
C321.ΔA.564	*C321.ΔA.* $endA^-$ gor^- $mazF^-$ rna^- rnb^-	This study
C321.ΔA.563	*C321.ΔA.* $endA^-$ $mazF^-$ rna^- rnb^- rne^-	This study
**Plasmids**		
pY71-sfGFP	Km^R, P_{T7}::super folder green fluorescent protein (sfGFP), C-terminal strep-tag	[20]
pY71-sfGFP-E132	pY71-sfGFP with amber codon at E132	[20]
pY71-sfGFP-T216	pY71-sfGFP with amber codon at T216	[20]
pY71-sfGFP-2UAG	pY71-sfGFP with amber codon at N212 and T216	[21]
pY71-sfGFP-5UAG	pY71-sfGFP with amber codon at D36, K101, E132, D190, and E213	[21]
pY71-sfGFP-8tdUAG	pY71-sfGFP with 8 consecutive amber codons at E132	This study
pY71-sfGFP-9tdUAG	pY71-sfGFP with 9 consecutive amber codons at E132	This study
pY71-CAT	Km^R, P_{T7}::chloramphenicol acetyl transferase (CAT)	[2]
pY71-CAT-D112UAG	pY71-CAT with amber codon at D122	[2]
pK7-mGM-CSF	Km^R, P_{T7}::modified murine granulocyte-macrophage colony-stimulating factor (mGM-CSF)	[22]
pK7-DHFR	Km^R, P_{T7}::dihydrofolate reductase (DHFR)	New England Biolabs
pY71-pAcFRS	P_{T7}::pAcFRS, C-terminal 6x histidine tag	[21]
pY71-pPaFRS	P_{T7}::pPaFRS, C-terminal 6x histidine tag	[20]
pDAK-pAzFRS	P_{T7}::pAzFRS, C-terminal 6x histidine tag	This study
pY71-mRFP-Spinach	P_{T7}::mRFP-Spinach aptamer	[23]
pDULE-o-tRNA	Tet^R, P_{lpp}::o-tRNA	[20]
pEVOL-pAcF	Cm^R, P_{glnS}::*pAcFRS*, P_{araBAD}::*pAcFRS*, P_{proK}::o-$tRNA^{opt}$	[24]
pY71-T7-tz-o-$tRNA^{opt}$	P_{T7}:: hammer-head ribozyme (tz), o-$tRNA^{opt}$ (o-tz-tRNA)	[21]
pY71-KA-sfGFP	N-ter KpnI and C-ter ApaI restriction site addition on sfGFP	This study
pY71-ELP-20	ELP-20mer	This study
pY71-ELP-30	ELP-30mer	This study
pY71-ELP-40	ELP-40mer	This study
pY71-ELP-20UAG	ELP-20mer with 20 amber sites	This study

pY71-ELP-30UAG	ELP-30mer with 30 amber sites	This study
pY71-ELP-40UAG	ELP-40mer with 40 amber sites	This study
pY71-ELP-20-TS	ELP-20mer with Twin-Streptag	This study
pY71-ELP-30-TS	ELP-30mer with Twin-Streptag	This study
pY71-ELP-40-TS	ELP-40mer with Twin-Streptag	This study
pY71-ELP-20UAG-TS	ELP-20mer with 20 amber sites, Twin-Streptag	This study
pY71-ELP-30UAG-TS	ELP-30mer with 30 amber sites, Twin-Streptag	This study
pY71-ELP-40UAG-TS	ELP-30mer with 40 amber sites, Twin-Streptag	This study

Supplementary Table 4. Doubling times of MAGE engineered strains. Cells were grown in 2xYTPG medium at 34 ˚C in a 96-well plate. Percentage relative to *C321.ΔA* is shown. Each data point is the average of nine replicates from three independent cultures (*n*=3, biological replicates). For each condition, error bar = 1 standard deviation.

Strains	Doubling time (min)	Percentage (%)
BL21 Star (DE3)	33.0 ± 0.0	96
C321.ΔA	34.2 ± 0.7	100
C321.ΔA.540	40.0 ± 0.7	117
C321.ΔA.541	34.0 ± 0.3	99
C321.ΔA.542	35.6 ± 0.3	104
C321.ΔA.598	33.2 ± 0.9	97
C321.ΔA.618	34.0 ± 1.2	99
C321.ΔA.620	37.0 ± 0.7	108
C321.ΔA.626	37.2 ± 0.6	109
C321.ΔA.628	35.0 ± 0.3	102
C321.ΔA.644	35.6 ± 0.7	104
C321.ΔA.666	35.2 ± 0.3	103
C321.ΔA.667	40.8 ± 0.6	119
C321.ΔA.668	37.4 ± 0.9	109
C321.ΔA.669	40.0 ± 1.2	117
C321.ΔA.672	34.0 ± 0.3	99
C321.ΔA.674	33.6 ± 0.6	98
C321.ΔA.544	34.0 ± 0.3	99
C321.ΔA.678	37.2 ± 0.6	109
C321.ΔA.679	35.8 ± 0.9	105
C321.ΔA.709	37.6 ± 1.2	110
C321.ΔA.711	35.6 ± 0.3	104
C321.ΔA.708	43.5 ± 1.3	127
C321.ΔA.703	38.0 ± 1.2	111
C321.ΔA.705	41.2 ± 2.5	120
C321.ΔA.706	38.8 ± 3.0	113
C321.ΔA.740	38.0 ± 0.3	111
C321.ΔA.738	41.4 ± 1.2	121
C321.ΔA.759	44.4 ± 1.6	130

Supplementary Table 5. CFPS yields for additional MAGE engineered strains. Active sfGFP cell-free protein synthesis (CFPS) yields from 12 distinct extracts, each derived from a MAGE-generated mutant in this study. Fold changes are relative to CFPS yields obtained from extracts derived from *C321.ΔA*. Three independent CFPS reactions for each sample were performed (*n*=3). Error bar = 1 standard deviation.

Strain	Active yield (μg/mL)			Fold change
C321.ΔA	350	±	6	1.0
C321.ΔA.619	36	±	4	0.1
C321.ΔA.617	410	±	14	1.2
C321.ΔA.621	37	±	1	0.1
C321.ΔA.680	850	±	16	2.4
C321.ΔA.664	640	±	6	1.8
C321.ΔA.756	1,340	±	70	3.8
C321.ΔA.755	700	±	35	2.0
C321.ΔA.758	1,250	±	71	3.5
C321.ΔA.879	940	±	62	2.7
C321.ΔA.878	850	±	84	2.4
C321.ΔA.564	600	±	27	1.7
C321.ΔA.563	540	±	12	1.5

Supplementary Table 6. Accumulated polymorphisms in engineered strains. A summary of point mutations in engineered extract chassis strains based on whole genome sequencing. Both intentional and off-target polymorphisms are included. Two clones of strain *C321.ΔA* 759 were sequenced.

Strain	Total Polymorphisms	Polymorphisms in ORFs	Polymorphisms introduced via MAGE
C321.ΔA.542	24	21	1
C321.ΔA.705	46	39	3
C321.ΔA.709	31	22	2
C321.ΔA.740	51	41	3
*C321.ΔA.759*_1	52	44	4
*C321.ΔA.759*_2	52	43	4

Supplementary Table 7. Genetic loci with polymorphisms in engineered strains. Listed are open reading frames featuring mutations in engineered extract chassis strains based on whole genome sequencing. Both intentional and off-target polymorphisms are included. Nucleotide coordinates listed are relative to the C321 genome previously submitted to NCBI.[19] Two clones of strain *C321.ΔA* 759 were sequenced. Mutations in MAGE-targeted loci are in bold.

C321.ΔA.542	***C321.ΔA.705***	***C321.ΔA.709***	***C321.ΔA.740***	***C321.ΔA.759_1***	***C321.ΔA.759_2***
mhpB	yaaU	yaaU	19780	yaaU	yaaU
glnQ	leuO	cueO	yaaU	leuO	leuO
rlmC	secA	glnQ	leuO	mmuP	mmuP
1126557	mmuP	yliI	secA	294012	294012
stfE	294012	890768	294012	brnQ	brnQ
pinR	brnQ	rlmC	mhpC	mdlB	mdlB
ydeO	mdlB	ybjT	mdlB	glnQ	glnQ
ynfB	nfrA	1126557	entS	yliI	yliI
yoaE	glnQ	1205553	zitB	rlmC	890763
mdtC	yliI	ydeO	glnQ	ybjT	rlmC
yeiP	rlmC	ynfB	yliI	1126557	ybjT
2699606	ybjT	1823360	rlmC	**rne**	1126557
recD	1126557	yoaE	911037	1356014	**rne**
xanQ	**rne**	yeiP	ybjT	fdnG	1356014
**endA**	1205553	focB	macB	ydeO	fdnG
yhcM	1356014	2771468	ycaI	ynfB	ydeO
nirB	fdnG	xanQ	1075484	ydiI	ynfB
bglB	ydeO	**endA**	1126557	ydjN	ydiI
yigB	ynfB	murA	1205606	yoaE	ydjN
4040864	yoaE	yhcM	stfE	2083525	yoaE
bdcR	2083525	nirB	insZ	yeiP	2083525
insO	yeiP	**gor**	pinR	hisQ	yeiP
sgcC	hisQ	bglB	paaH	focB	hisQ
yjjX	upp	3910303	ydeO	upp	focB
	2745789	3984603	ynfB	yfjH	upp
	yfjH	yigB	yoaE	2771468	yfjH
	gutM	4040864	2157207	gutM	2771468
	hypE	4411975	yeiP	hypE	gutM
	xanQ	insO	yfdK	**mazF**	hypE
	**endA**	sgcC	focB	xanQ	**mazF**
	rpoD	yjjX	yfhM	**endA**	xanQ
	yqjH		yfjH	rpoD	**endA**
	ebgR		2771468	yqjH	rpoD
	yhcD		hypF	ebgR	yqjH
	yhcM		**mazF**	nusA	ebgR
	gspG		xanQ	yhcD	nusA
	nirB		**endA**	yhcM	yhcD

gph	trmI	nirB	yhcM
**gor**	glcD	gph	nirB
bglB	obgE	**gor**	gph
xerC	yhcM	bglB	**gor**
yigB	**gor**	3984603	mdtF
4040864	dppC	xerC	bglB
insO	malS	yigB	3984603
sgcC	bglB	4040863	xerC
yjjX	3984603	4411975	yigB
	yigB	ulaF	4040864
	4040864	ytfI	4411975
	insO	insO	ulaF
	sgcC	insI1	insO
	yjjX	sgcC	sgcC
		yjjX	yjjX

Supplementary Methods

Strain construction and verification

The strains in this study (**Supplementary Table 3**) were generated from *C321.ΔA*[19] by disrupting genes of interest (**Supplementary Table 1**) with mutagenic oligonucleotides via MAGE (**Supplementary Table 2**). Cultures were grown in LB-Lennox media (10 g/L tryptone, 5 g/L yeast extract and 5 g/L NaCl) at 32 ˚C and 250 rpm throughout MAGE cycling steps[25]. MAGE oligonucleotides were designed to introduce an internal stop codon and frameshift ~1/4 of the way into the target gene sequence thereby causing early translational termination as previously reported[2]. Combinatorial disruptions of *endA*, *mazF*, *rna*, *rnb*, *rne*, *gor*, *lon*, *ompT*, *gdhA*, *gshA*, *sdaA*, *sdaB*, *speA*, *tnaA*, and *glpK* were generated to investigate the effects of their inactivation on CFPS. Multiplex allele-specific colony (MASC) PCR was performed to verify gene disruptions using wild-type forward (-wt-F) or mutant forward (-mut-F) primers and reverse primers (-R) (**Supplementary Table 2**)[25]. Wild-type and mutant forward primers were identical except at the 3'-ends of the oligonucleotides, and the reverse primers were used for detection of both wild-type and mutant alleles. The mutant allele could be amplified using the mutant forward and reverse primer set (-mut-F and -R) but not amplified by the wild-type forward and reverse primer set (-wt-F and -R). MASC PCR was performed in 10 µL reactions using a multiplex PCR kit (Qiagen, Valencia, CA). Mutant alleles were screened by running PCR products on a 1.5% agarose gel and confirmed by DNA sequencing using sequencing primers (**Supplementary Table 2**).

Tandem UAG plasmid construction

Eight and nine tandem UAG sequences were added into the sfGFP at position 132 by inverse PCR followed by ligation. The resulting plasmids are pY71-sfGFP-8tdUAG and -9tdUAG (**Supplementary Table 3**).

ELP plasmid construction

ELP genes were codon optimized for *E. coli* expression. ELPs contained three pentapeptides in a monomer unit for 20, 30, and 40 -mers and an amber site in a monomer unit for ncAA incorporation (**Fig. 4a**)[26]. First, we constructed the pY71-KA-sfGFP vector by adding KpnI and ApaI restriction sites at the 5'- and 3'-end of the sfGFP gene, respectively (**Supplementary Table 2**). ELP genes were cloned into this vector using the same restriction sites resulting in ELPs with C-terminal sfGFP fusion. Then, we added a Twin-Streptag using ApaI and BlpI restriction sites in place of the sfGFP gene (**Supplementary Table 2**). All ELP plasmids are listed in **Supplementary Table 3**.

Growth rate assessment

Overnight cultures of engineered strains grown in Luria-Bertani (LB) at 250 rpm at 34 ˚C were diluted 1000-fold in 2xYTPG media (31 g/L 2xYT, 7 g/L K_2HPO_4, 3 g/L KH_2PO_4, and 18 g/L glucose; adjusted pH to 7.2 with KOH)[27]. 100 µL of the diluted cultures were added to 96-well polystyrene plates (costar 3370; Corning, Corning, NY). OD was measured at 15 min intervals for 15 h at 34 ˚C in fast shaking mode on a Synergy 2 plate reader (Biotek, Winooski, VT). Growth data of each strain was obtained from three independent cultures, each split into three replicate wells (9 total samples per strain). Doubling time was calculated during the early exponential growth phase (OD_{600} of 0.02 to 0.2).

Cell extract preparation

To make cell extract, cell pellets were thawed and suspended in 0.8 mL of S30 buffer per gram of wet cell mass and 1.4 mL of cell slurry was transferred into 1.5 mL microtubes. The cells were lysed using a Q125 Sonicator (Qsonica, Newtown, CT) with a 3.175 mm diameter probe at a 20 kHz frequency and 50% amplitude. To minimize heat damage during sonication, samples were placed in an ice-water bath. For each 1.4 mL sample, the input energy during sonication was monitored and ceased at ~944 Joules. The extract was then centrifuged at 12,000 g at 4 ˚C for 10 min. For strain derivatives of MG1655, a run-off reaction (37 ˚C at 250 rpm for 1 h) and second centrifugation (10,000 g at 4 ˚C for 10 min) were performed[28]. The supernatant was flash-frozen using liquid nitrogen and stored at –80 ˚C until use. The

total protein concentration of the extracts was 40 to 50 mg/mL as measured by Quick-Start™ Bradford protein assay kits (Bio-Rad, Hercules, CA).

Purification of His-tagged orthogonal tRNA synthetase

BL21 (DE3) harboring a pY71 plasmid encoding either pAcFRS, pAzFRS, or pPaFRS were grown in 1 L of 2xYT to an OD_{600} of 1.0 at 220 rpm and 37 ˚C. Orthogonal synthetase production was induced by adding 0.3 mM isopropyl-β-D-thiogalactopyranoside (Sigma-Aldrich, St. Louis, MO) and cells were allowed to grow for an additional 3 h. Cells were harvested at 5,000 g for 15 min at 4 ˚C, washed with S30 buffer, and stored at −80 ˚C. Frozen cell pellets were thawed in loading buffer (1 mL of 300 mM NaCl, 10 mM imidazole, 50 mM NaH_2PO_4, pH 8.0 solution per gram of wet cells), lysed using sonication as described above and centrifuged at 16,000 g at 4 ˚C for 10 min. The supernatant was diluted 1:1 with loading buffer and incubated at 4 ˚C for 1 h with Ni-NTA beads prewashed with dilution buffer (300 mM NaCl, 50 mM NaH_2PO_4, pH 8.0). The orthogonal synthetase was purified using elution buffer (300 mM NaCl, 250 mM imidazole, 50 mM NaH_2PO_4, pH 8.0) and subsequently dialyzed against S30 buffer and 25% glycerol in a Slide-A-Lyzer™ G2 Dialysis Cassette (Life Technology, Grand Island, NY). Dialyzed synthetase was concentrated using an Amicon Ultracel YM-30 centrifugal filter and stored at −80 ˚C. Purified synthetase was quantified by the Quick-Start™ Bradford protein assay kit (Bio-Rad, Hercules, CA).

Reagents and chemicals

Carbenicillin (50 μg/mL) was used for culturing *C321.ΔA* derivative strains, kanamycin (50 μg/mL) was used for maintaining pY71-based plasmids, and chloramphenicol (34 μg/mL) was used to maintain the pEVOL-pAcF plasmid. The *E. coli* total tRNA mixture (from strain MRE600) and phosphoenolpyruvate was purchased from Roche Applied Science (Indianapolis, IN). ATP, GTP, CTP, UTP, 20 amino acids

and other materials were purchased from Sigma-Aldrich (St. Louis, MO) without further purification. T7 RNA Polymerase was purified in house using ion exchange chromatography as described previously[29].

Fed-batch CFPS reactions

For fed-batch reactions, 15 μL CFPS batch reactions were prepared as described above. At the specified time, the reactions were removed from the incubator, supplied with 0.5 μL of feeding solution containing the appropriate concentration of the desired amino acid(s), thoroughly mixed with a pipette, and returned to the incubator. All reactions were incubated at 30 ˚C for a total of 20 h and assayed.

Scale-up CFPS reactions

Cell-free reaction volumes were scaled up to 255 μL in flat-bottom 24-well polystyrene plate (model 353226; BD Biosciences, San Jose, CA). Remaining wells around the perimeter of the plate were filled with water for internal humidification, which resulted in reduced sample evaporation. Reactions were performed at 30 ˚C for 20 h while shaking at 300 RPM in a ThermoMixer (Eppendorf, Mississauga, Ontario). Sufficient sfGFP and ELP were purified for mass spectrometry analysis.

Quantification of active sfGFP

Active full-length sfGFP protein yields were quantified by measuring fluorescence using a Synergy 2 plate reader (BioTek, Winooski, VT) with excitation at 485 nm, emission at 528 nm, and cut-off at 510 nm in 96-well half area black plates (Costar 3694; Corning, Corning, NY). sfGFP fluorescence units were converted to concentration using a standard curve established with ^{14}C-Leu quantified sfGFP as described previously[21].

Quantification of total and soluble protein

Radioactive ^{14}C-Leucine was added into 15 μL CFPS reactions at a final concentration of 10 μM. For reactions synthesizing ELPs, ^{14}C-Glycine was used. Reactions were taken at the indicated time and 5 μL

of sample was removed for total protein quantitation. The remaining sample was centrifuged at 16,000 g at 4 ˚C for 10 min and the top 5 µL was used to measure the soluble protein. Total and soluble protein yields were quantified by determining radioactive ^{14}C-Leu incorporation into trichloroacetic acid (TCA) - precipitated protein[30]. The radioactivity of TCA-precipitated samples was measured using liquid scintillation counting (MicroBeta2, PerkinElmer, Waltham, MA).

Autoradiogram analysis

For autoradiogram analysis, 2 µL of each reaction was loaded on a 10% NuPAGE SDS-PAGE gel after denaturing the sample. The gel was soaked in Gel Drying solution (Bio-Rad, Hercules, CA) for 30 min, fixed with cellophane films, dried overnight in a GelAir Dryer (Bio-Rad, Hercules, CA), and exposed for 3 days on a Storage Phosphor Screen (GE Healthcare Biosciences, Pittsburgh, PA). Autoradiograms were scanned using a Storm Imager (GE Healthcare Biosciences, Pittsburgh, PA).

Whole genome analysis

Because the gene encoding MutS is inactivated in *C321.ΔA*, we chose to fully sequence the genomes of six key strains produced during our screening efforts (*C321.ΔA*, *C321.ΔA.542*, *C321.ΔA.705*, *C321.ΔA.709*, *C321.ΔA.740*, *C321.ΔA.759*). One milliliter of cell culture in LB broth was processed with a Qiagen DNeasy Blood and Tissue kit (cat: 69504) to extract genomic DNA (gDNA). gDNA quality was assessed on a spectrophotometer (assay for A260/280 ratio between 1.8 and 2.0) and by gel electrophoresis (assay for a tight smear at 50 kB). 2.5 µg of gDNA, eluted in 50-µl TE pH 8.0, was sent to the Yale Center Genome Analysis for library prep and analysis as described previously[31]. Genome modification of targeted effectors were confirmed and off target point mutations were limited to regions that were not expected to effect CFPS activity.

mRNA stability assay

mRNA stability was assessed as described previously[2]. Briefly, the sfGFP gene was PCR amplified from the pY71 vector and purified using a PCR clean-up kit (Promega, Madison, WI). This template was used for T7-driven *in vitro* transcription reactions. In order to track mRNA stability in our extracts, sfGFP was synthesized using purified mRNA (1,800 ng) in the CFPS reaction. For direct measurement of mRNA degradation, purified mRNA samples from the cell-free reaction were visualized on a 2% formaldehyde agarose gel stained with GelRed (Biotium, Hayward, CA). mRNA band intensities were quantified using ImageJ software (National Institutes of Health, Bethesda, MD) and normalized to rRNA bands.

DNA stability assay

DNA stability was assessed as described previously[2]. Briefly, a pre-incubation mixture containing 4 μL of cell extract, 12.96 ng/μL of pY71-mRFP1-Spinach plasmid (**Supplementary Table 3**), and 67 μM of 3,5-difluoro-4-hydroxybenzylidene imidazolinone (DFHBI; Lucerna, New York, NY) was incubated for 0, 60, and 180 min at 30 ˚C. CFPS reaction components were added immediately after the pre-incubation step, and fluorescence of the Spinach aptamer binding to DFHBI was monitored for 180 min using a CFX96 Real-Time RT-PCR module installed on a C1000 Touch Thermal Cycler (Bio-Rad, Hercules, CA). The excitation and emission wavelengths of the fluorophore were 450-490 nm and 515-530 nm, respectively.

Nucleotide and amino acid quantitation using HPLC

Amino acid and nucleotide concentrations were measured via HPLC. For amino acid analysis, a Poroshell HPH-C18 column (4.6 x 100 mm, 2.7 μm particle size; Agilent, Santa Clara, CA) was used with an automatic pre-column derivatization method using *o*-pthalaldehyde (OPA) and fluorenylmethyl chloroformate (FMOC)[32]. Run times were 16 min at a flow rate of 1.5 mL/min and a column temperature of 40 ˚C. Mobile phase A was comprised of 10 mM sodium phosphate, 10 mM sodium tetraborate, and 5 mM sodium azide at pH 8.2. Mobile phase B was 45% methanol, 45% acetonitrile, and 10% water by volume. The buffer gradient for B was: 0 min, 2%; 0.5 min, 2%; 13.4 min, 57%; 13.6 min, 100%; 15.7

min, 100%; 15.9 min, 2%; 16 min, end. All amino acids were detected at 262 nm except for proline, which was detected at 338 nm. Concentrations were determined by comparison to a standard calibration using NIST standard reference material 2389a. Amino acids not contained in the NIST standard were obtained from Sigma-Aldrich.

Nucleotides were analyzed using a BioBasic AX column (4.6 x 150 mm, 5 µm particle size; Thermo Scientific, West Palm Beach, FL). Separation was carried out at a flow rate of 1 mL/min and column temperature of 22 ˚C. Mobile phase A and B respectively were 5 and 750 mM potassium phosphate monobasic adjusted to pH 3.30 with phosphoric acid. The buffer gradient for B was: 0 min, 0%; 6 min, 20%; 11 min, 40%; 20 min, 100%; 25 min, 100%; 25.5 min, 0%; 30 min, end. Nucleotides were detected at 254 nm. Concentrations were determined by comparison to a standard calibration.

Full-length sfGFP and ELP purification and mass spectrometry

To confirm pAcF incorporation at corresponding amber sites, liquid chromatography mass spectrometry (LC-MS) analysis was performed on purified sfGFP and ELP reporter proteins. First, full-length sfGFP protein was purified from CFPS reactions using C-terminal strep-tags and a 0.2 mL gravity-flow Strep-Tactin Sepharose mini-columns (IBA GmbH, Gottingen, Germany) and concentrated using Microcon YM-10 centrifugal filter columns (Millipore, Billerica, MA). ELPs were purified using a modified inverse transition cycling (ITC) method in which cell-free reactions were centrifuged at 14,000 g for 3 min at room temperature to capture aggregated ELPs in the pellet. The isolated pellet was then resuspended in cold 1xPBS solution to resolubilize the ELP. The resulting mixture was then centrifuged at 14,000 g for 5 min at 4 ˚C. To precipitate the ELPs, sodium citrate was added to the mixture at a final concentration of 0.5 M and the resulting mixture was centrifuged at 14,000 g for 3 min at room temperature to capture aggregated ELPs in the pellet. These steps were repeated as necessary to purify ELP from contaminants.

Purified reporter protein was then injected onto a trap column (150 μm ID × 3 cm) coupled with a nanobore analytical column (75 μm ID × 15 cm). The trap and analytical column were packed with polymeric reverse phase (PLRP-S, Phenomenex, Torrance, CA) media (5 μm, 1,000 Å pore size). Samples were separated using a linear gradient of solvent A (95% water, 5% acetonitrile, 0.2% formic acid) and solvent B (5% water, 95% acetonitrile, 0.2% formic acid). Samples were loaded for 10 min onto the trap and were subsequently separated using a linear gradient from 5% to 55% of solvent B over 27 min followed by washing steps. Mass spectrometric data were obtained on a 12T Velos FT Ultra (Thermo-Scientific) instrument fitted with a custom nanospray ionization source. The acquisition method was comprised of three scan events occurring sequentially and repeatedly throughout the course of the protein elution. The first scan event was a full scan FTMS experiment, where data was obtained from 500-2000 Da at a resolving power of 170,000 at m/z 400. For the second scan event, a data-dependent tandem MS experiment was performed in which the most intense ion in the FTMS spectrum was fragmented (resolving power 50,000; isolation width 15; normalized collision energy 41; activation Q 0.4; activation time 100 ms). Scan event three was another full scan experiment in which mass measurement occurred in the Velos ion trap. FTMS data were deconvoluted using Xtract (ThermoFisher) and Protein Deconvolution 4.0 and average masses were reported. In the MS figures (**Fig. 3c, Fig. 4d-f,** and **Supplementary Fig. 11**), smaller peaks ($\Delta m = +16$ Da) are due to oxidation of the protein – a common electrochemical reaction occurring during electrospray ionization. The presence of the initial methionine amino acid residue on a protein will also increase the mass ($\Delta m = +131$ Da), which we detected in some samples. To remove non-covalent salt and water adducts from intact proteins (in this case sfGFP), a small level of in-source collision energy (15V) was applied. As a result, water loss events from the intact sfGFP ($\Delta m = -18$ Da) are detected at minor levels to the left of the major (colored) peak.

Supplementary References

1. Atkinson DE. The energy charge of the adenylate pool as a regulatory parameter. Interaction with feedback modifiers. *Biochemistry* **7**, 4030-4034 (1968).

2. Hong SH*, et al.* Improving cell-free protein synthesis through genome engineering of *Escherichia coli* lacking release factor 1. *Chembiochem* **16**, 844-853 (2015).

3. Michel-Reydellet N, Woodrow K, Swartz J. Increasing PCR fragment stability and protein yields in a cell-free system with genetically modified Escherichia coli extracts. *J Mol Microbiol Biotechnol* **9**, 26-34 (2005).

4. Borja GM, Meza Mora E, Barron B, Gosset G, Ramirez OT, Lara AR. Engineering Escherichia coli to increase plasmid DNA production in high cell-density cultivations in batch mode. *Microb Cell Fact* **11**, 132 (2012).

5. Zhang Y, Zhang J, Hoeflich KP, Ikura M, Qing G, Inouye M. MazF cleaves cellular mRNAs specifically at ACA to block protein synthesis in *Escherichia coli*. *Molecular cell* **12**, 913-923 (2003).

6. Raines RT. Ribonuclease A. *Chem Rev* **98**, 1045-1066 (1998).

7. Airen IO. Genome-wide funtional genomic analysis for physiological investigation and improvement of cell-free protein synthesis. In: *Stanford University* (Palo Alto, CA). Stanford University (USA) (2011).

8. Kushner SR. mRNA decay in Escherichia coli comes of age. *J Bacteriol* **184**, 4658-4665; discussion 4657 (2002).

9. Prud'homme-Genereux A, Beran RK, Iost I, Ramey CS, Mackie GA, Simons RW. Physical and functional interactions among RNase E, polynucleotide phosphorylase and the cold-shock protein, CsdA: evidence for a 'cold shock degradosome'. *Molecular microbiology* **54**, 1409-1421 (2004).

10. Bundy BC, Swartz JR. Efficient disulfide bond formation in virus-like particles. *J Biotechnol* **154**, 230-239 (2011).

11. Jiang XP, Oohira K, Iwasaki Y, Nakano H, Ichihara S, Yamane T. Reduction of protein degradation by use of protease-deficient mutants in cell-free protein synthesis system of Escherichia coli. *J Biosci Bioeng* **93**, 151-156 (2002).

12. Goerke AR, Loening AM, Gambhir SS, Swartz JR. Cell-free metabolic engineering promotes high-level production of bioactive Gaussia princeps luciferase. *Metabolic Engineering* **10**, 187-200 (2008).

13. Grabowska A, Nowicki M, Kwinta J. Glutamate dehydrogenase of the germinating triticale seeds: gene expression, activity distribution and kinetic characteristics. *Acta Physiol Plant* **33**, 1981-1990 (2011).

14. Calhoun KA, Swartz JR. Total amino acid stabilization during cell-free protein synthesis reactions. *J Biotechnol* **123**, 193-203 (2006).

15. Michel-Reydellet N, Calhoun K, Swartz J. Amino acid stabilization for cell-free protein synthesis by modification of the Escherichia coli genome. *Metab Eng* **6**, 197-203 (2004).

16. Lin ECC. Glycerol Dissimilation and Its Regulation in Bacteria. *Annu Rev Microbiol* **30**, 535-578 (1976).

17. Rittmann D, Lindner SN, Wendisch VF. Engineering of a glycerol utilization pathway for amino acid production by *Corynebacterium glutamicum*. *Appl Environ Microb* **74**, 6216-6222 (2008).

18. Wang HH*, et al.* Programming cells by multiplex genome engineering and accelerated evolution. *Nature* **460**, 894-898 (2009).

19. Lajoie MJ*, et al.* Genomically recoded organisms expand biological functions. *Science* **342**, 357-360 (2013).

20. Bundy BC, Swartz JR. Site-specific incorporation of p-propargyloxyphenylalanine in a cell-free environment for direct protein-protein click conjugation. *Bioconjugate Chemistry* **21**, 255-263 (2010).

21. Hong SH, Ntai I, Haimovich AD, Kelleher NL, Isaacs FJ, Jewett MC. Cell-free protein synthesis from a release factor 1 deficient Escherichia coli activates efficient and multiple site-specific nonstandard amino acid incorporation. *ACS Synth Biol* **3**, 398-409 (2014).

22. Yang J, Kanter G, Voloshin A, Levy R, Swartz JR. Expression of active murine granulocyte-macrophage colony-stimulating factor in an Escherichia coli cell-free system. *Biotechnol Prog* **20**, 1689-1696 (2004).

23. Fritz BR, Jewett MC. The impact of transcriptional tuning on *in vitro* integrated rRNA transcription and ribosome construction. *Nucleic Acids Research* **42**, 6774-6785 (2014).

24. Young TS, Ahmad I, Yin JA, Schultz PG. An enhanced system for unnatural amino acid mutagenesis in E. coli. *J Mol Biol* **395**, 361-374 (2010).

25. Wang HH, Church GM. Multiplexed genome engineering and genotyping methods applications for synthetic biology and metabolic engineering. *Methods Enzymol* **498**, 409-426 (2011).

26. Amiram M*, et al.* Evolution of translation machinery in recoded bacteria enables multi-site incorporation of nonstandard amino acids. *Nat Biotechnol* **33**, 1272-1279 (2015).

27. Sambrook J, Fritsch, E.F., and Maniatis, T. *Molecular Cloning, A Laboratory Manual, Cold Spring Harbor Laboratory Press. ColdSS Spring Harbor, NY* (1989).

28. Kwon YC, Jewett MC. High-throughput preparation methods of crude extract for robust cell-free protein synthesis. *Sci Rep* **5**, 8663 (2015).

29. Jewett MC, Swartz JR. Mimicking the Escherichia coli cytoplasmic environment activates long-lived and efficient cell-free protein synthesis. *Biotechnol Bioeng* **86**, 19-26 (2004).

30. Swartz JR, Jewett MC, Woodrow KA. Cell-free protein synthesis with prokaryotic combined transcription-translation. *Methods Mol Biol* **267**, 169-182 (2004).

31. Gallagher RR, Patel JR, Interiano AL, Rovner AJ, Isaacs FJ. Multilayered genetic safeguards limit growth of microorganisms to defined environments. *Nucleic Acids Research* **43**, 1945-1954 (2015).

32. Henderson JW, Brooks, A. Improved Amino Acid Methods using Agilent ZORBAX Eclipse Plus C18 Columns for a Variety of Agilent LC Instrumentation and Separation Goals. (Agilent Technologies, Application Note, Santa Clara, CA) (2010)
